# Phase relationships in homoleptic com­plexes of XeF_2_

**DOI:** 10.1107/S2052252526003751

**Published:** 2026-05-18

**Authors:** Lewis A. Clough, Kristian Radan, Dominik Daisenberger, Joseph Hriljac, Nico Giordano, Matic Lozinšek, Simon Parsons

**Affiliations:** a EaStCHEM School of Chemistry and Centre for Science at Extreme Conditions University of Edinburgh King's Buildings West Mains Road Edinburgh EH9 3FJ United Kingdom; b Diamond Light Source Harwell Science and Innovation Campus Didcot Oxfordshire OX11 0DE United Kingdom; c Extreme Conditions Chemistry Laboratory Jožef Stefan Institute Jamova cesta 39 1000 Ljubljana Slovenia; d Deutsches Elektronen-Synchrotron DESY 22607 Hamburg Germany; Sun Yat-Sen University, China

**Keywords:** high-pressure crystallography, high-pressure phase transitions, low-tem­per­a­ture phase relationships, noble-gas chemistry

## Abstract

The structural behaviour of homoleptic xenon difluoride (XeF_2_) com­plexes [*M*(XeF_2_)_6_][SbF_6_]_2_ (*M* = Cu, Zn) under varying tem­per­a­ture and pressure has been investigated, aiming to resolve the disordered Jahn–Teller distortions in the copper com­plex (**CuSb**). At 200 K, both **CuSb** and its zinc analogue (**ZnSb**) crystallize in a layered CdCl_2_-type structure with the space group *R*3. Upon cooling below 170 (**CuSb**) and 160 K (**ZnSb**), both systems transition to iso­structural phases in *P*1, with **CuSb** assuming an ordered Jahn–Teller distortion. The transformation is driven by the shortening and optimization of the Xe⋯F inter­molecular contacts, forming stronger and more directional inter­actions, rather than by Jahn–Teller effects alone. This is supported by the observation of similar transitions in the Jahn–Teller-inactive Zn system. High-pressure ex­peri­ments up to ∼2.8 GPa at room tem­per­a­ture show the structural stability of the high-symmetry phases, implicating kinetic barriers to further transformation. Additionally, the synthesis and structural characterization of a novel arsenic analogue, [Zn(XeF_2_)_6_][AsF_6_]_2_ (**ZnAs**), reveal similar layered motifs but distinct phase behaviour. Symmetry-mode analyses relate all observed phases through distortions of a common CdCl_2_ aristotype.

## Abstract 2

The com­pounds [*M*(XeF_2_)_6_][*A*F_6_]_2_, where *M* = Cu or Zn and *A* = As or Sb, contain unusual homoleptic com­plexes of XeF_2_ which form a series of different phases under varying conditions of tem­per­a­ture and pressure. The phases are related to the CdCl_2_ aristotype and are connected through symmetry-lowering reorientations of the cations and anions.

## 1. Introduction

The synthesis and characterization of the three binary fluorides of xenon, *i.e.* XeF_2_ (Chernick *et al.*, 1962 ▸; Hoppe *et al.*, 1962 ▸; Weeks *et al.*, 1962 ▸), XeF_4_ (Claassen *et al.*, 1962 ▸; Chernick *et al.*, 1962 ▸; Slivnik *et al.*, 1962*a* ▸) and XeF_6_ (Malm *et al.*, 1963 ▸; Weaver *et al.*, 1963 ▸; Slivnik *et al.*, 1962*b* ▸), were described within only a year of Bartlett’s first synthesis of a noble-gas com­pound (Bartlett, 1962 ▸). Two com­pounds containing both XeF_6_ and KrF_2_, [F_5_Xe(FKrF)AsF_6_] and [F_5_Xe(FKrF)_2_AsF_6_], have been reported recently (Lozinšek *et al.*, 2021 ▸). XeF_2_ can act as a fluoride-ion donor to metal centres in coordination com­plexes (Brock *et al.*, 2013 ▸). The coordination chemistry of KrF_2_ has also been investigated, though it is more limited than that of XeF_2_.

Homoleptic com­plexes with noble-gas fluoride ligands are com­paratively rare, and are observed only for Ca^2+^, Ni^2+^, Cu^2+^ and Zn^2+^ with XeF_2_, and for Hg^2+^ with KrF_2_ (De Backere & Schrobilgen, 2018 ▸). A metal centre in a homoleptic coordination environment of XeF_2_ was first reported in [Ca_2_(XeF_2_)_9_(AsF_6_)_4_] (Tramšek *et al.*, 2004 ▸), followed by the syntheses and crystal structures of [*M*(XeF_2_)_6_][SbF_6_]_2_ (*M* = Cu, Zn; Scheme 1, *A* = Sb) (Tavčar *et al.*, 2006 ▸), with [*M*(XeF_2_)_6_][RuF_6_]_2_ (*M* = Ni, Cu) being reported recently (Mržljak *et al.*, 2025 ▸). [Cu(XeF_2_)_6_][RuF_6_]_2_ crystallizes in the space group *P*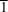, with each Cu centre sitting on an inversion centre and exhibiting a Jahn–Teller distortion, with Cu—F bond lengths between 1.907 (4) and 2.167 (5) Å. By contrast, the Cu and Zn com­plexes with [SbF_6_]^−^, as well as [Ni(XeF_2_)_6_][RuF_6_]_2_, crystallize in the space group *R*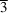, with the metal occupying a site such that all ligands have equal *M*—F bond lengths. This suggests either a dynamic Jahn–Teller distortion or static disorder in copper com­plexes (Deeth & Hitchman, 1986 ▸; Bersuker, 2021 ▸).
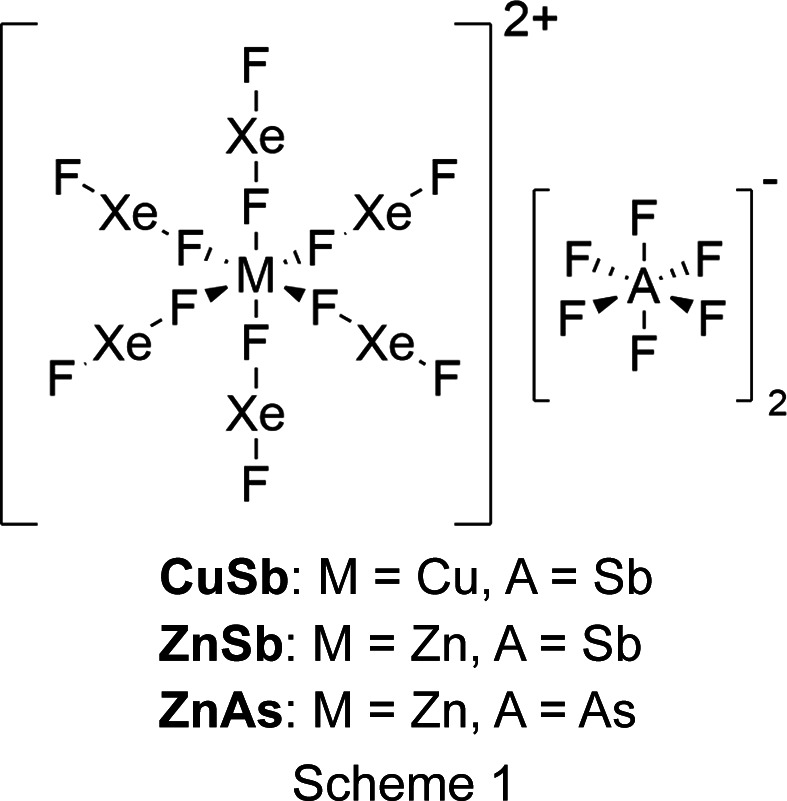


Disorder is an entropy-driven process which arises in crystal structures when there is sufficient thermal energy to access multiple thermodynamically com­petitive configurations. One approach for resolving dynamically disordered structures into ordered forms is to reduce the tem­per­a­ture. Examples of Jahn–Teller ordering in Cu^2+^ chemistry include [Cu{(C_5_H_4_N)_3_CH}_2_][NO_3_]_2_, which shows only one Cu—N EXAFS peak at 295 K, but two at 150 K (Astley *et al.*, 1995 ▸). Likewise, the Cu—N bond lengths in [Cu(*L*)_2_][BF_4_]_2_ [*L* = 2,6-bis­(pyrazol-1-yl)pyridine] [Cambridge Structural Database (CSD; Groom *et al.*, 2016 ▸) refcode PEFQIA] gradually converge on raising the tem­per­a­ture from 31 to 350 K, the structure transforming from *Z*′ = 3 to *Z*′ = 1 at 41 K (Beddard *et al.*, 2003 ▸).

Since (∂*S*/∂*P*)_*T*_ = −α*V* (where α is the thermal expansion coefficient, *V* the volume and *T* the tem­per­a­ture) is negative for most materials, another mechanism for reducing the effect of entropy *S* is to increase the pressure *P*. Pressure has been used to resolve dynamic Jahn–Teller effects, but this has been less extensively studied than the use of tem­per­a­ture. In (Ph_4_P)_2_IC_60_ (Francis *et al.*, 2012 ▸), the dynamic distortion exhibited by the C_60_^−^ anion is resolved to a static distortion at 2 GPa, as identified by the splitting of degenerate vibrational modes. Although application of pressure to resolve Jahn–Teller disorder in copper com­plexes appears not to have been investigated, successive pressure-induced switching of the Jahn–Teller distortions along the N—Cu—N, O—Cu—O and F—Cu—F axes has been observed in the coordination polymer [CuF_2_(H_2_O)_2_(pyrazine)_2_]_2_ (Halder *et al.*, 2011 ▸).

**Figure 1:**
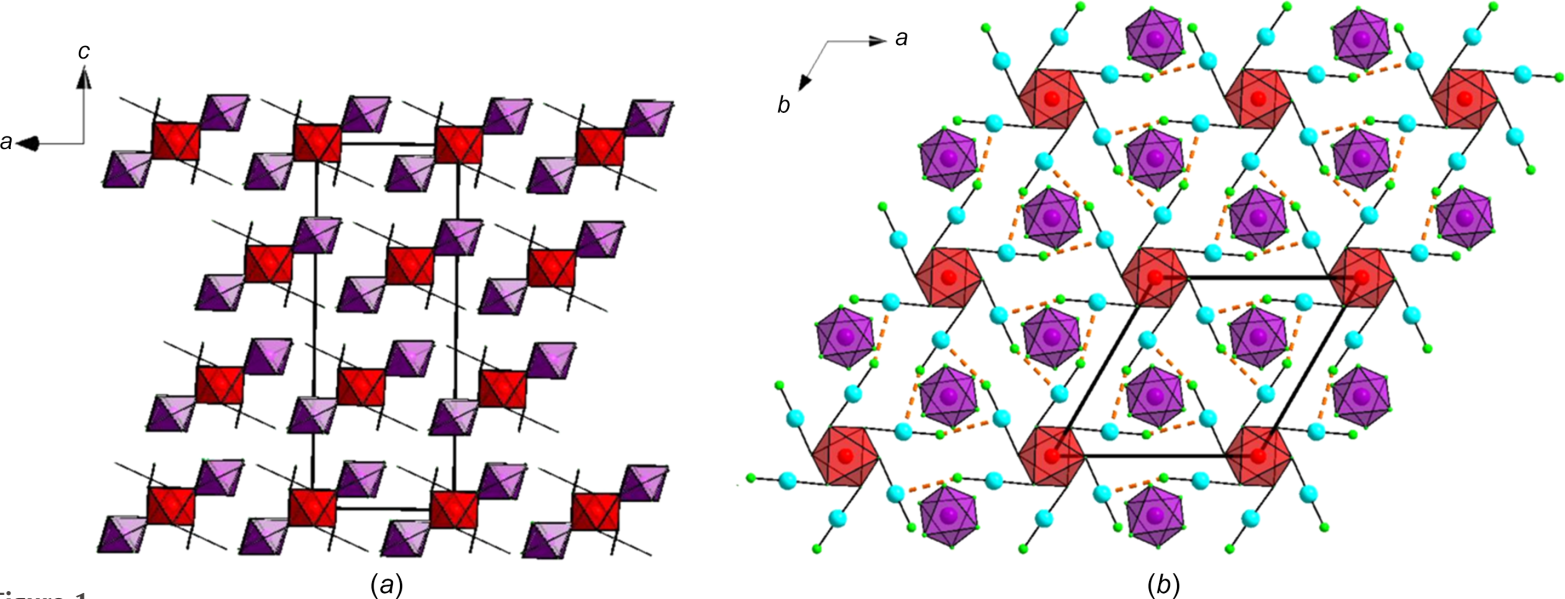
Packing in the crystal structure of **CuSb-I**. (*a*) View along [010] showing the stacking of the layers along the *c* axis. (*b*) The layers viewed along [001], with the short intra­layer Xe—F⋯Xe cation–cation contacts repre­sent­ed by dashed bonds. The colour scheme here and in the other figures is red for Cu and purple for Sb. XeF_2_ ligands are shown in ‘stick’ format in part (*a*).

This article aims to explore the effect of tem­per­a­ture and pressure on the Jahn–Teller disorder in [Cu(XeF_2_)_6_][SbF_6_]_2_ (**CuSb**, Fig. 1 ▸). The Xe atoms in **CuSb** and all other xenon com­pounds show a strong tendency to form short directional contacts to atoms bearing lone pairs, especially fluorine. The directionality of these contacts has been investigated using theoretical methods (Kirshenboim & Kozuch, 2016 ▸; Gomila & Frontera, 2020 ▸) and, as we show below, a number of them in the ambient-tem­per­a­ture disordered structure of **CuSb** can be regarded as non-optimal. Thus, although it is highly moisture sensitive and somewhat difficult to prepare and handle, this material is an ideal candidate for exploring the capacity of external conditions to reduce the effects of entropy and resolve disorder in crystal structures by driving the selection of optimal inter­molecular inter­actions.

The article also describes parallel experiments on the analogous and structurally related zinc-containing systems [Zn(XeF_2_)_6_][SbF_6_]_2_ (**ZnSb**) and [Zn(XeF_2_)_6_][AsF_6_]_2_ (**ZnAs**). The Zn atom in these com­pounds is not Jahn–Teller active and the results provide a control for defining the role of the Jahn–Teller effect itself in driving the phase transitions described.

## 2. Experimental

### 2.1. Synthetic procedures

#### 2.1.1. Sources of materials

XeF_2_ was synthesized *via* the photosynthetic method from F_2_ (Solvay Fluor, 98–99%) and Xe (Messer, 99.99%) (Šmalc & Lutar, 1992 ▸). ZnF_2_ (99.995%) and CuF_2_ (99.5%) were ob­tained from Thermo Fisher Scientific. Hydrogen fluoride (Linde, 99.995%) was treated with K_2_NiF_6_ (Advance Re­search Chemicals, 99.9%) prior to use. Halocarbon oil 11-14 (Halocarbon Products Corp.), Fomblin Z60 and Fomblin Z25 (Synquest Laboratories) were used as pressure-transmitting media (Motaln *et al.*, 2025 ▸). Perfluoro­deca­lin was used as supplied from Sigma–Aldrich (batch number STBH2228). *M*(SbF_6_)_2_ (*M* = Cu, Zn) were prepared from the corresponding metal difluorides, SbF_3_ (Sigma–Aldrich, 99.8%) and excess F_2_ in anhydrous HF (Gantar *et al.*, 1987 ▸).

**Caution!** Anhydrous HF (aHF), F_2_, XeF_2_, AsF_5_, SbF_5_ and their com­pounds must be handled with extreme care in a well-ventilated fume hood, with appropriate protective equipment worn at all times.

#### 2.1.2. Synthesis of [*M*(XeF_2_)_6_][SbF_6_]_2_ (*M* = Cu or Zn; CuSb and ZnSb)

The salts [*M*(XeF_2_)_6_][SbF_6_]_2_ (*M* = Cu or Zn) were synthesized from XeF_2_ and the corresponding *M*(SbF_6_)_2_ salt in aHF [Equation (1)] (Tavčar *et al.*, 2006 ▸). An h-shaped FEP reaction-crystallization vessel equipped with a PCTFE valve was assembled (see supporting information, Fig. S1) and exposed to *ca* 0.5 atm (1 atm = 101325 Pa) of elemental fluorine for 12 h, followed by evacuation under dynamic vacuum for 3 h prior to use. In the case of the zinc derivative, Zn(SbF_6_)_2_ (58.2 mg, 0.108 mmol) was combined with XeF_2_ (173.4 mg, 1.02 mmol) in the reaction branch of the vessel under nitro­gen. aHF (*ca* 1.5 ml) was condensed into the vessel at 77 K, before gentle warming and stirring to dissolve the solids. The colourless solution was stirred for 12 h, before transfer to the crystallization arm of the vessel. A tem­per­a­ture gradient of 5–10 K was applied between the arms of the vessel to promote slow evaporation of aHF over a period of six weeks, resulting in a batch of colourless crystals. These were cooled to 230 K and dried under dynamic vacuum. The Cu derivative was prepared in a similar manner using Cu(SbF_6_)_2_ (76.4 mg, 0.143 mmol) and XeF_2_ (231.1 mg, 1.36 mmol).

Crystalline reaction products were identified as a mixture of [*M*(XeF_2_)_6_][SbF_6_]_2_ and [Xe_2_F_3_][SbF_6_] [Equations (1) and (2); *M* = Cu or Zn] using single-crystal X-ray diffraction. [*M*(XeF_2_)_6_][SbF_6_]_2_ were apparently the minor products based on random sampling of the visually indistinguishable crystals.
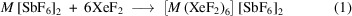

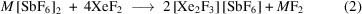


#### 2.1.3. Synthesis of [Zn(XeF_2_)_6_][AsF_6_]_2_ (ZnAs)

XeF_2_ (273.1 mg, 1.61 mmol) was combined with ZnF_2_ (21.7 mg, 0.21 mmol) in a similar reaction vessel to that described in Section 2.1.2. aHF (*ca* 1.7 ml) was condensed into the reaction vessel at 77 K, before gentle warming and stirring to dissolve the solids. AsF_5_ (74.8 mg, 0.44 mmol) was condensed into the reaction vessel gradually in six approximately equal aliquots, warming and stirring the solution to ensure full dissolution of AsF_5_ between additions. Warming to room tem­per­a­ture yielded a pale-yellow solution which was stirred for 12 h. Crystallization, as described in Section 2.1.2, yielded pale-yellow crystals. [Zn(XeF_2_)_6_][AsF_6_]_2_ [**ZnAs**, Equa­tion (3)] and [Xe_2_F_3_][AsF_6_] [Equation (4)] were identified as the crystalline products by single-crystal X-ray dif­fraction, with the dominant phase found being the homoleptic com­plex.
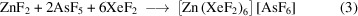

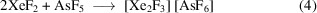


Attempted synthesis of [Cu(XeF_2_)_6_][AsF_6_]_2_ from CuF_2_ using a similar procedure was unsuccessful, with the only detected reaction products being [Xe_2_F_3_][AsF_6_], CuF_2_ and XeF_2_.

### 2.2. X-ray diffraction data collection and processing

#### 2.2.1. Low-tem­per­a­ture single-crystal X-ray diffraction

Single crystals of **ZnAs**, **ZnSb** and **CuSb** were selected under perfluoro­deca­lin and mounted on a Bruker AXS D8 Venture diffractometer equipped with an Oxford Cryosystems low-tem­per­a­ture device operating at 200 K. Diffraction data were collected between 200 and 80 K in increments of 10 K using Ag or Mo *K*α radiation (λ = 0.56083 and 0.71073 Å, respectively) generated using Incoatec microsources. A further data set was collected at 30 K for **CuSb** using an Oxford Cryosystems N-Helix low-tem­per­a­ture device. Cooling induced transitions to new phases in both **CuSb** and **ZnSb**. Different phases are distinguished using Roman numerals, *i.e.***CuSb-I**, **CuSb-II**, *etc.* The variation of unit-cell volume with tem­per­a­ture is shown in Fig. S2 and Tables S1–S3 of the supporting information.

The data were processed in *APEX5* (Bruker, 2024 ▸) and integrated using *SAINT*. Corrections for absorption and other systematic errors were applied using the multi-scan procedures in *SADABS* or *TWINABS* (Krause *et al.*, 2015 ▸). The structures were solved using dual-space methods (*SHELXT*; Sheldrick, 2015*a* ▸) and refinement of all structures was against |*F*|^2^ using *SHELXL* (Sheldrick, 2015*b* ▸) from within the *OLEX-2* inter­face (Dolomanov *et al.*, 2009 ▸) or *CRYSTALS* (Betteridge *et al.*, 2003 ▸).

With the exception of **CuSb-I**, all datasets were affected by twinning or the presence of multiple domains. The sample of **ZnSb-I** was split into two domains related by a 115.7° rotation about [0
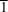
1]. Data were integrated over both domains and those reflections containing a contribution from the major domain used in the structure refinement. The domain scale factor refined to 0.278 (2). **CuSb-II** was twinned *via* a 120° rotation about [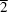
10] but also split. The reference domain was dominant (estimated scale factor ∼0.9) and the most suc­cessful data reduction strategy was to ignore the twinning during integration and then apply the twin law to those data where the maximum deviation from integral values in the transformed Miller indices was 0.25 or less. The scale factor refined to 0.0651 (15). Diffraction data for **ZnSb-II** contained contributions from at least eight different domains. The first domain was dominant and, after some experimentation, only the second twin domain (generated by a twofold rotation about [100]) was included in the integration, but refinements against the ‘detwinned’ dataset output by *TWINABS* proved to give the best agreement statistics. Data for **ZnAs-I** were <!?tlsb=-0.1pt>integrated over three domains generated by 180 and 93.5° rotations about [101]. All three domains were used in the structure analysis, with final domain scale factors of 0.4578 (14) and 0.0757 (14), respectively. Further details of the twin handling are available in the CIFs in the supporting information.

A non-standard setting of the unit cell of **ZnAs-I** was used to facilitate com­parison with **ZnSb-II**.

#### 2.2.2. High-pressure single-crystal X-ray diffraction

Crystals suitable for high-pressure single-crystal diffraction were selected under nitro­gen in a glovebox equipped with a Nikon SMZ1500 video microscope, which has an operating distance suitable for manipulation of diamond anvil cells (DACs). The DACs were of a miniature Merrill–Bassett (Merrill & Bassett, 1974 ▸) or a mini-BX80 design (Kantor *et al.*, 2012 ▸), both with half-opening angles of 38° and fitted with Boehler–Almax type II diamond anvils of culet diameter 600 or 700 µm ob­tained from Almax easyLab (Boehler & De Hantsetters, 2004 ▸). The sample chamber was constructed using rhenium gaskets of thickness 200 µm indented in different experiments to between 75 and 110 µm with holes of diameter 300 and 500 µm drilled by spark erosion. The materials studied are highly oxidizing, and we observed differing sensitivities to the pressure-transmitting media reported by Motaln *et al.* (2025 ▸). The final structures reported were ob­tained using Halocarbon Oil 11-14 for **ZnAs**, Fomblin Z25 for **CuSb** and Z60 for **ZnSb**. Pressure was determined using the ruby fluorescence method (Shen *et al.*, 2020 ▸).

High-pressure X-ray diffraction data were recorded for **CuSb** and **ZnSb** at the Extreme Conditions Beamline (P02.2) at DESY (Liermann *et al.*, 2015 ▸) using synchrotron radiation of wavelength 0.2906 Å, and for **ZnAs** at Diamond Light Source I-15 Extreme Conditions Beamline using radiation of wavelength 0.1582 Å. Pressure was increased in steps of ∼0.5 GPa up to approximately 2.78 GPa; beyond this pressure, the quality of the diffraction patterns had deteriorated and they could not be indexed reliably. Data were processed using *CrysAlis PRO* (Rigaku, 2023 ▸). Corrections for absorption and gasket shading were applied using the multi-scan method (Blessing, 1995 ▸; Rigaku, 2023 ▸). Structures were solved and refined as described above, the starting model at each pressure point was taken from the previous point in the series. All atomic positions were refined with anisotropic displacement parameters (ADPs) subject to enhanced rigid-bond restraints (Thorn *et al.*, 2012 ▸). The structure of **ZnAs-II** required an additional restraint in which As2—F and As4—F distances were restrained to 1.72 (2) Å, and F⋯F distances restrained to 2.43 (2) Å to maintain an octa­hedral geometry. Equivalent distance and ADP similarity restraints were also used to maintain reasonable bond lengths and ADPs.

Listings of selected crystal and refinement data are available in Tables 1 ▸–3 ▸ ▸, with data for all structures available in CIF format in the supporting information. Structures were visualized using *Mercury* (Macrae *et al.*, 2020 ▸) or *DIAMOND* (Version 4; Putz & Brandenberg, 2024 ▸). Structure analysis was performed using *PLATON* (Spek, 2020 ▸). Continuous shape measures were calculated using *SHAPE* (Alvarez *et al.*, 2002 ▸; Cirera *et al.*, 2005 ▸).

## 3. Results

### 3.1. [Cu(XeF_2_)_6_][SbF_6_]_2_ (CuSb) structures

#### 3.1.1. CuSb-I

At 200 K, **CuSb** crystallizes in the space group *R*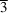 (the hexa­gonal setting was used throughout), with *Z* = 3 and *Z*′ = 0.167; we shall refer to this phase as **CuSb-I** [Figs. 1 ▸, 2 ▸(*a*) and 3 ▸(*a*)]. The structure consists of discrete [Cu(XeF_2_)_6_]^2+^ cations with six equivalent linear monodentate XeF_2_ ligands bound to Cu1 through F1. The Xe1—F1 bond distance is slightly longer than the terminal Xe1—F2 distance at 2.087 (2) and 1.929 (3) Å, respectively, as is common for coordinating XeF_2_ ligands (Tramšek & Žemva, 2006 ▸). The Cu atoms occupy the lattice points of the rhombohedral unit cell. This 3*a* site has 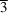 (*S*_6_) symmetry and all the Cu—F bond lengths [1.9877 (19) Å] are equal, with apparently no Jahn–Teller distortion. Xe1⋯F1 contacts of 3.442 (3) and 3.470 (2) Å are formed between ligands attached to the same Cu^2+^ centre. The octa­hedral [SbF_6_]^−^ anions occupy the 6*c* sites and have crystallographic 3 (*C*_3_) point symmetry.

**Figure 2:**
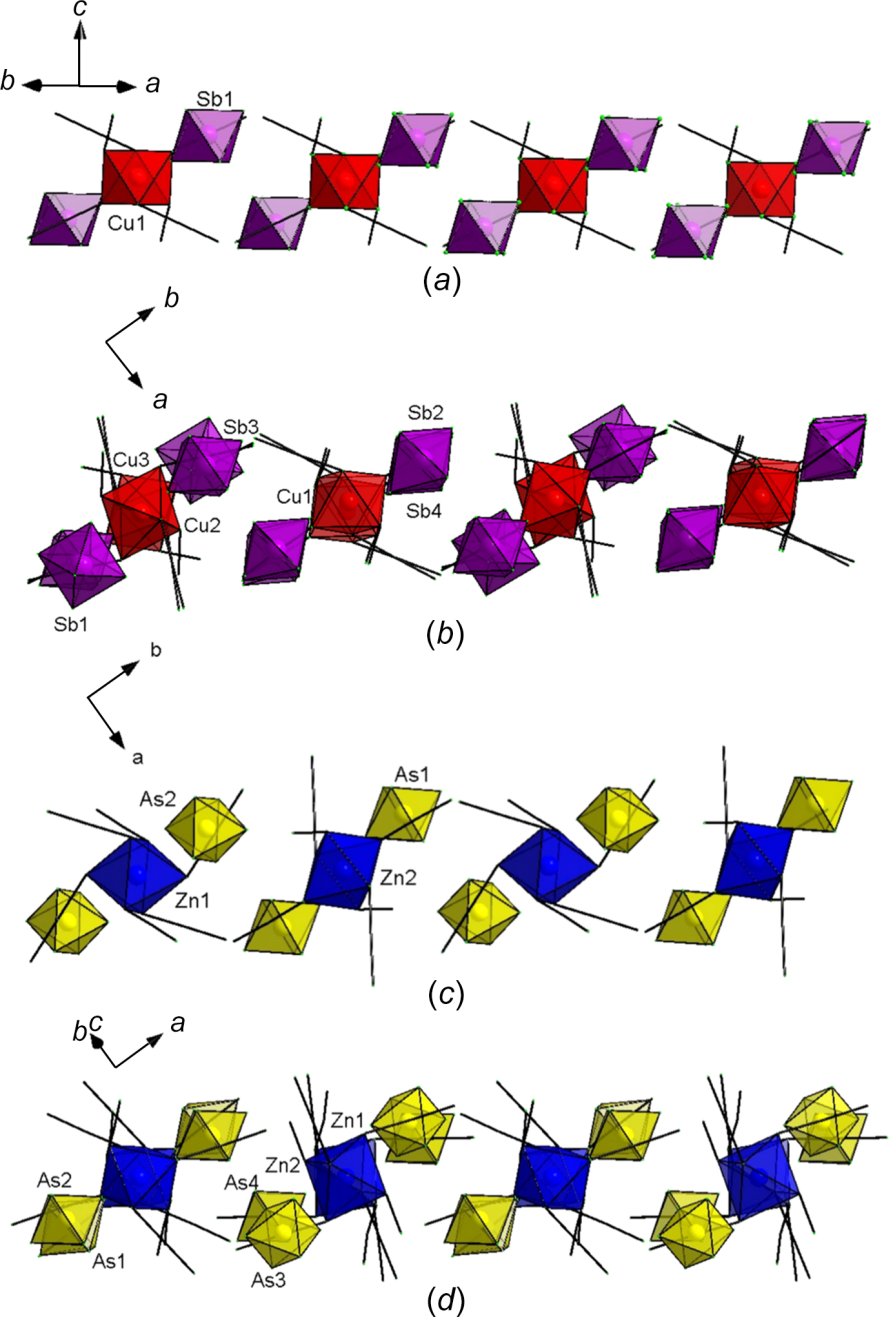
The structures of layers in the **CuSb** and **ZnAs** phases, showing (*a*) **CuSb-I**, (*b*) **CuSb-II**, (*c*) **ZnAs-I** and (*d*) **ZnAs-II**. The figures are projected down the axis that best highlights their similarity to CdCl_2_. The colours in parts (*a*) and (*b*) are as in Fig. 1 ▸. In parts (*c*) and (*d*), Zn polyhedra are shown in blue and As in yellow. XeF_2_ ligands are shown in ‘stick’ format.

The packing in **CuSb-I** resembles that in CdCl_2_ (Fig. S3). The Cd and Cl atoms occupy positions with the same fractional coordinates in CdCl_2_ as Cu and Sb in **CuSb-I**, but the space group symmetry of CdCl_2_ is reduced by the XeF_2_ and fluoride ligands. Both structures consist of layers which are stacked along the *c* axes of the unit cells (Fig. 1 ▸). The layers [Fig. 2 ▸(*a*)] themselves are com­posed of a layer of cations sandwiched between two layers of anions. The direct chloride bridging of the CdCl_2_ structure is replaced in **CuSb-I** by cation–anion inter­actions mediated by Xe⋯F contacts. The anions are located adjacent to triangular faces of the CuXe_6_ octa­hedra, with pairs of F⋯Xe contacts made to two Xe atoms forming one edge of the octa­hedron [two F3⋯Xe1 contacts measuring 3.549 (4) and 3.571 (4) Å to one Xe1, and F3⋯Xe1 and F4⋯Xe1 contacts measuring 3.474 (3) and 3.694 (4) Å to the other; the sum of the van der Waals radii of Xe and F is 3.74 Å (Vogt & Alvarez, 2014 ▸)]. Substanti­ally shorter F2⋯Xe1 contacts measuring 3.182 (4) Å are formed to Xe atoms of the cations in the second coordination shell of the same layer, and these decorate the upper and lower surfaces of the layers [Fig. 1 ▸(*b*)].

The contacts between the layers com­prise short F2⋯Xe1 contacts measuring 3.211 (2) Å involving pairs of cations, and longer F4⋯Xe1 inter­actions [3.565 (4) Å] between cations and anions.

The mol­ecular electrostatic potential of XeF_2_ shows a positive belt around the equator of the mol­ecule (Kirshenboim & Kozuch, 2016 ▸; Gomila & Frontera, 2020 ▸). The maximum value of the potential is not located perpendicular to the F—Xe—F axis, but instead there are two maxima, which are slightly displaced towards the F atoms. These lie between the accumulations of electron density associated with the Xe—F bonding and Xe-based lone pairs anti­cipated from the Valence Shell Electron Pair Repulsion (VSEPR) model. Theoretical models suggest that optimal contacts usually subtend angles between 60 and 75° to the primary Xe—F bonds (Gomila & Frontera, 2020 ▸). This is true for the shortest Xe1⋯F2 contacts [∠F2—Xe1⋯F2 = 75.39 (10) and 64.27 (11)°], as well as the longer contacts to F3 and F4 [69.63 (9) and 68.13 (12)°, respectively]. Other contacts are formed in directions which are nearly perpendicular to the Xe—F bonds, for example, the F1—Xe1⋯F3 and F4 contacts measuring 3.549 (4) and 3.565 (4) Å form angles of 82.89 (8) and 87.83 (11)° at Xe1.

#### 3.1.2. CuSb-II

At 170 K, **CuSb-I** undergoes a transition to a triclinic phase, **CuSb-II**, in the space group *P*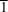, with *Z* = 4 and *Z*′ = 2. No further structural transitions occur on cooling to 30 K. The increase in *Z*′ from 0.167 to 2 leads to a substantial increase in structural com­plexity [Figs. 2 ▸(*b*) and 3(*b*)–(*d*)]. The matrix relating the basis vectors of the unit cell of **CuSb-I** (**a**_I_, *etc*.) to those of **CuSb-II** (**a**_II_, *etc*.) is given in Equation (5).
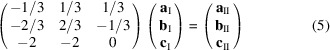


The integrity of the sample is preserved across the transition, but the threefold axis of rotation, which was present along [001] in phase I becomes a twin law about [2
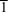
0] in phase II.

The asymmetric unit of **CuSb-II** contains one [Cu(XeF_2_)_6_]^2+^ cation on a general position (based on Cu1) and two more located on inversion centres (Cu2 and Cu3). Cu1 is bound to six crystallographically unique XeF_2_ ligands based on Xe11 to Xe61, which bind to Cu1 through odd-numbered F atoms F11, F31,…F111; the terminal F atoms carry even-based labels, F21, F41,…F121. The labelling in the cations based on Cu2 and Cu3 is similar, except that the Xe and F labels extend to Xe32, F62, Xe33 and F63 as a result of the inversion symmetry. There are four [SbF_6_]^−^ anions in general positions, based on Sb1–Sb4, respectively, carrying F14–F64 to F17–F67.

The averaged Jahn–Teller distortion of phase I is resolved into a static distortion in phase II. Around Cu1, atoms F11 and F91 are extended to distances of 2.100 (6) and 2.099 (7) Å, while the other Cu1—F bonds are between 1.920 (6) (Cu1—F51) and 2.004 (6) Å (Cu1—F71). Similarly, Cu2 exhibits elongated Cu2—F32 bonds [2.095 (6) Å] and shortened Cu2—F12 and Cu2—F52 bonds [1.924 (6) and 1.961 (6) Å, respectively]. Cu3 has long Cu3—F53 bonds [2.114 (6) Å] and short Cu3—F13 and Cu3—F33 bonds [1.931 (6) and 1.932 (6) Å, respectively]. The average Cu—F distance is 1.998 Å, which is very similar to that in **CuSb-I**. The effect of the Jahn–Teller distortion is propagated to the ligands, a long Cu—F bond leading to shorter (Cu)F—Xe bonds, which have an average length of 2.058 (4) Å com­pared to 2.106 (3) Å for the remainder.

**CuSb-II** is, like **CuSb-I**, based on the CdCl_2_ structure. The layered packing arrangement is retained, but while the cation based on Cu1 remains in a similar orientation to phase I, Cu2 and Cu3 both undergo rotation within the layer [Figs. 2 ▸(*b*) and 3(*b*)–(*d*)]. The anions based on Sb1 and Sb3 have both undergone significant reorientation, while those based on Sb2 and Sb4 maintain more similar orientations to the phase I structure. An animation of the transition, calculated using symmetry mode analysis (*ISODISTORT*) (Campbell *et al.*, 2006 ▸; Stokes *et al.*, 2025 ▸) for a simplified structure consisting only of the [CuF_6_]^2+^ and [SbF_6_]^−^ octa­hedra is available in the supporting information (Movie_1). This demonstrates the large and small reorientations which have occurred in alternate columns consisting of cations and anions running along the *c* direction of phase II. The loss of alignment of the different com­ponents of the full structure can be seen by com­parison of Figs. 2(*a*) ▸ and 2(*b*) ▸.

The movie referred to in the previous paragraph was gen­er­a­ted by linear inter­polation along the irreducible representations which govern the transition, and it should not be inter­preted as depicting a mechanism. Nevertheless, the symmetry analysis does enable the transformation to be broken down into a formal sequence of symmetry-lowering steps, shown in Fig. 4 ▸ in the form of a Bärnighausen tree (Müller, 2013 ▸), which helps to unravel the com­plexity of **CuSb-II**. The first step, labelled Γ_2_^+^Γ_3_^+^, generates the unit cell with metrics equal to the primitive rhombohedral setting of **CuSb-I**, but with the space-group symmetry reduced to *P*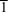 as a result of tilting of the octa­hedra away from the rotoinversion axes. The second generates a metrically monoclinic cell through excitation of the F_1_^+^ irreducible representation and leads to a doubling of the unit-cell volume. The effect is partially to reverse the tilting that occurred in the Γ_2_^+^Γ_3_^+^ step for some sites (those which become Cu1, Sb2 and Sb4 in phase II) and to accentuate it for the other sites, while retaining the translational symmetry along the [001] direction. This translational symmetry is broken in the third step, labelled Σ_1_, by rotating alternate Cu2, Sb1 and Sb3 octa­hedra clockwise and anti­clockwise about axes parallel to [001]; small alternating displacements occur for the Cu1, Sb2 and Sb3 octa­hedra. The combined effect is to generate **CuSb-II**, increasing the volume by another factor of two.

**Figure 3:**
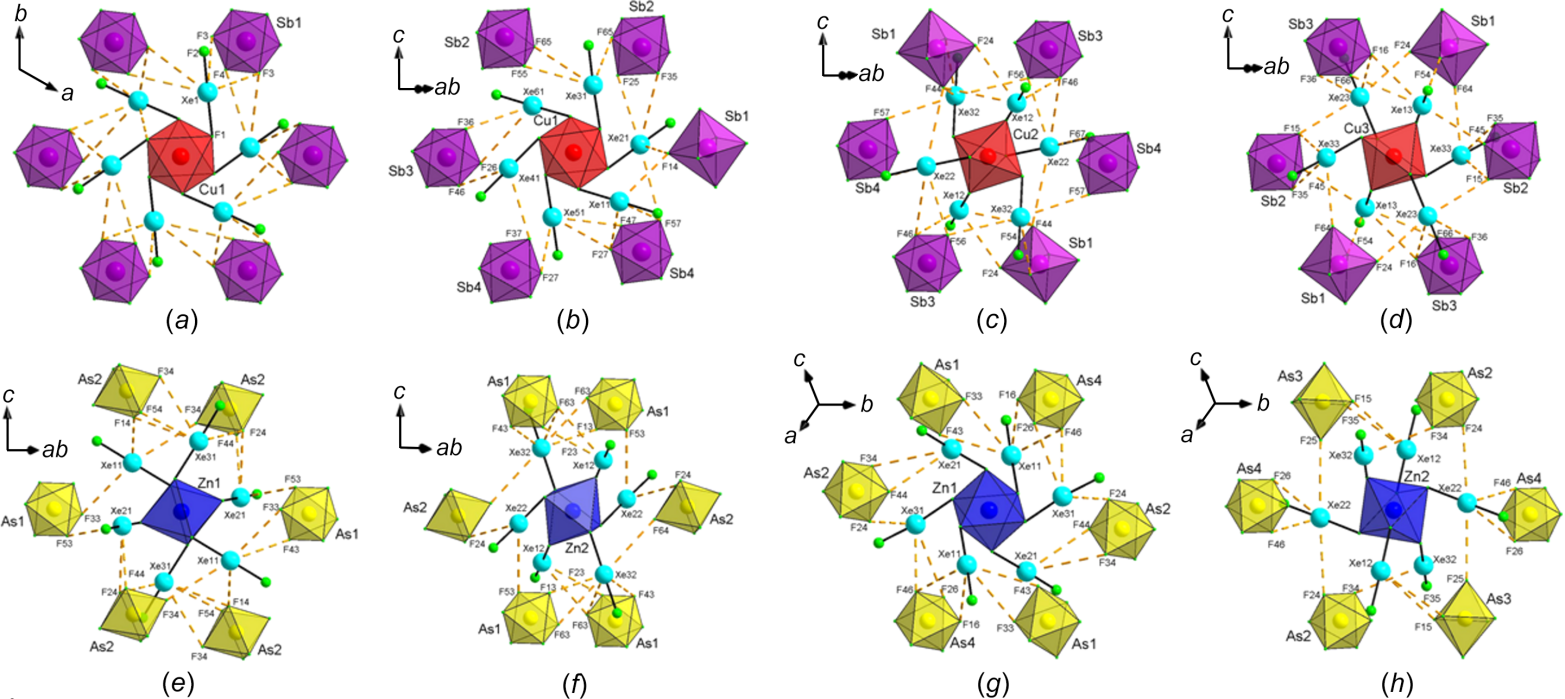
The Xe contacts between intra­layer anions and XeF_2_ ligands in (*a*) **CuSb-I**, (*b*) **CuSb-II** Cu1, (*c*) **CuSb-II** Cu2, (*d*) **CuSb-II** Cu3, (*e*) **ZnAs-I** Zn1, (*f*) **ZnAs-I** Zn2, (*g*) **ZnAs-II** Zn1 and (*h*) **ZnAs-II** Zn2. Inter­layer and same-centre contacts have been omitted for clarity.

The reorientations of the cations and anions result in reorganization of the Xe⋯F contacts. A notable feature of the structure of **CuSb-I** is that the shortest inter­molecular Xe⋯F contacts (<3.3 Å) are formed by the cations to other cations in the second mol­ecular coordination sphere, ‘leap-frogging’ the nearest-neighbour [SbF_6_]^−^ anions, which all form contacts exceeding 3.4 Å [Fig. 1 ▸(*b*)]. In phase II, there are many short Xe⋯F contacts involving the [SbF_6_]^−^ anions, for example, Xe11 and Xe33 have short intra­layer inter­actions of 3.168 (10) (F14) and 3.201 (8) Å (F64), respectively [Figs. 3 ▸(*b*) and 3(*d*)], with inter­layer contacts to F42 [3.309 (8) Å] and F61 [3.356 (9) Å]. Overall, the Xe⋯F contacts involving the anions span the range from 3.168 (10) (Xe11⋯F14) to 3.731 (8) Å (Xe21⋯F34), com­pared to a range from 3.474 (3) to 3.694 (4) Å in phase I.

The cation–cation contacts in phase II range between 3.063 (9) (Xe31⋯F101) and 3.697 (6) Å (Xe12⋯F52), com­pared to a range from 3.182 (4) to 3.470 (2) Å in phase I. The Xe⋯F contacts involving the anions generally shorten to a greater degree than those formed to cations: the maximum shortening exhibited by these contacts is 0.218 (6) Å for F12, com­pared to a maximum shortening of 0.306 (13) Å for the contacts to anions.

The inter­action angle ∠F—Xe⋯F is shown as a function of inter­action length for inter-ion contacts in Fig. 5 ▸. Points shown in blue correspond to phase I and those in red to phase II, the proliferation of the latter being a consequence of the lowering of symmetry. The distribution of square points in the plot makes clear the formation of short cation–anion contacts with optimal F—Xe⋯F angles in the range 60–75° over the course of the transition. By contrast, in phase I, the short cation–cation contacts (filled blue circles) all subtend optimal angles, but they are much more widely distributed in phase II, with some angles extending beyond 80° (filled red circles).

In phase I, the number of contacts making angles between 80 and 90° is 12 (both blue squares in Fig. 5 ▸ corresponding to six cation–anion inter­actions as a result of the high symmetry). In phase II, there are 13 such contacts, but these are now distributed between the Xe atoms in three crystallographically distinct cations. The average number of contacts beyond the optimal range is therefore 12 per Cu centre in phase I, but only 4.3 per Cu centre in phase II.

The transition from phase I to II is thus characterized by a strengthening in the cation–anion contacts, which are both shorter and formed at more optimal angles in phase II. At the same time, the disordered Jahn–Teller distortion of phase I becomes ordered in phase II. But what drives the transition? Is it an enthalpic effect associated with more optimal contact formation or the result of the reduced effect of entropy at low tem­per­a­ture? This question can be addressed by examining the isostructural Zn analogue, **ZnSb**.

### 3.2. [Zn(XeF_2_)_6_][SbF_6_]_2_ (ZnSb)

#### 3.2.1. ZnSb-I and ZnSb-II

**ZnSb** was, like **CuSb**, found in two phases, denoted **ZnSb-I** and **ZnSb-II. ZnSb-I**, which occurs above 160 K, is isostructural with **CuSb-I**. The Zn1—F1 bond length is 2.001 (5) Å. Minor differences in the inter­molecular contact distances are observed as a result of the difference in Cu—F and Zn—F bond lengths, though the ordering of inter-centre contacts remains the same as in **CuSb-I**.

**ZnSb-II**, which is formed at 160 K, is isostructural with **CuSb-II**. Zn^2+^ is Jahn–Teller inactive and the Zn—F bond lengths are more regularly distributed than in **CuSb-II**, ranging from 1.984 (6) to 2.021 (6) Å.

Both Cu^2+^ and Zn^2+^ have malleable coordination geometries (Gaazo *et al.*, 1976 ▸; Thomas *et al.*, 2023 ▸), and the principal significance of the results for **ZnSb** is that they clarify the role of changes in the geometry of the metal sites in the phase transitions. **CuSb** undergoes Jahn–Teller ordering on cooling, which causes the shape of the Cu coordination spheres to distort in phase II. By contrast, there are no significant changes in the Zn—F bond lengths over the course of the **ZnSb-I**→**ZnSb-II** transition (Table S3). Distortions in the shapes of the octa­hedra can be conveniently qu­anti­fied using continuous shape measures of the metal sites in **ZnSb-II** relative to that in **ZnSb-I** (Table S3) (Alvarez *et al.*, 2002 ▸; Cirera *et al.*, 2005 ▸). None of the values exceeds 0.06; indeed, the same is true for the other Zn-containing phases reported below. The transitions reported for the Zn system thus occur with minimal distortion to the coordination geometry and the transition seen in **CuSb** cannot be associated with the adoption of an ordered Jahn–Teller distortion in **CuSb-II**. The enthalpic effect of optimization of the cation–anion contacts is therefore the most likely driver for the phase transition.

### 3.3. High-pressure analysis of CuSb-I and ZnSb-I

The variation in unit-cell volume of **CuSb-I** and **ZnSb-I** between ambient pressure and 2.78 (5) GPa is shown in Fig. 6 ▸. The rhombohedral phase persisted for both com­pounds until diffraction quality had declined to a point where the patterns could not be indexed. Pressure therefore does not promote the symmetry-lowering transitions described above. The **CuSb** and **ZnSb** phase II structures have a smaller volume per formula unit than the phase I structures, and would be anti­cipated to be more stable at high pressure. It is possible that kinetic factors, such as the inhibition of ion rotation at high pressure, are the cause for the persistence of phase I.

The number of pressure points available for each com­pound is too limited to determine the equation-of-state parameters reliably, but as they exhibit very similar trends of volume *versus* pressure, an approximate average bulk modulus for the combined systems can be ob­tained. A second-order Birch–Murnaghan equation (Birch, 1947 ▸) yields an acceptable fit to the combined data, yielding a bulk modulus of 11.4 (6) GPa;, and the reference volume of the average Cu/Zn system refines to 1982 (10) Å^3^. The value of the bulk modulus is similar to the hy­dro­gen-bonded monoclinic phase of l-histidine [11.6 (6) GPa] (Novelli *et al.*, 2020 ▸) and l-alanine [13.1 (6) GPa] (Funnell *et al.*, 2010 ▸), as well as FeF_3_ [14 (1) GPa] (Jørgensen & Smith, 2006 ▸). The similarity of the trends depicted in Fig. 6 ▸ implies that the Jahn–Teller distortion present in **CuSb** plays little role in the com­pressibility mechanism.

### 3.4. [Zn(XeF_2_)_6_][AsF_6_]_2_ (ZnAs)

#### 3.4.1. ZnAs-I

The phases found for the **ZnAs** system are distinct from those of the [SbF_6_]^−^ salts described above. At ambient pressure between 100 and 200 K, the **ZnAs-I** phase forms in the space group *P*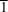, with *Z* = 2 and *Z*′ = 1. The structure is shown in Figs. 2 ▸(*c*) and 3(*e*)–(*f*). This is isostructural to the recently reported triclinic [Cu(XeF_2_)_6_][RuF_6_]_2_ phase at 100 K (Mržljak *et al.*, 2025 ▸).

There are two crystallographically independent Zn sites, both located on inversion centres, with Zn—F bond lengths between 1.974 (11) and 2.021 (11) Å. The [AsF_6_]^−^ anions lie on general positions. The structure has the same CdCl_2_ motif as the Sb-containing analogues [Fig. 2 ▸(*c*)].

**Figure 4:**
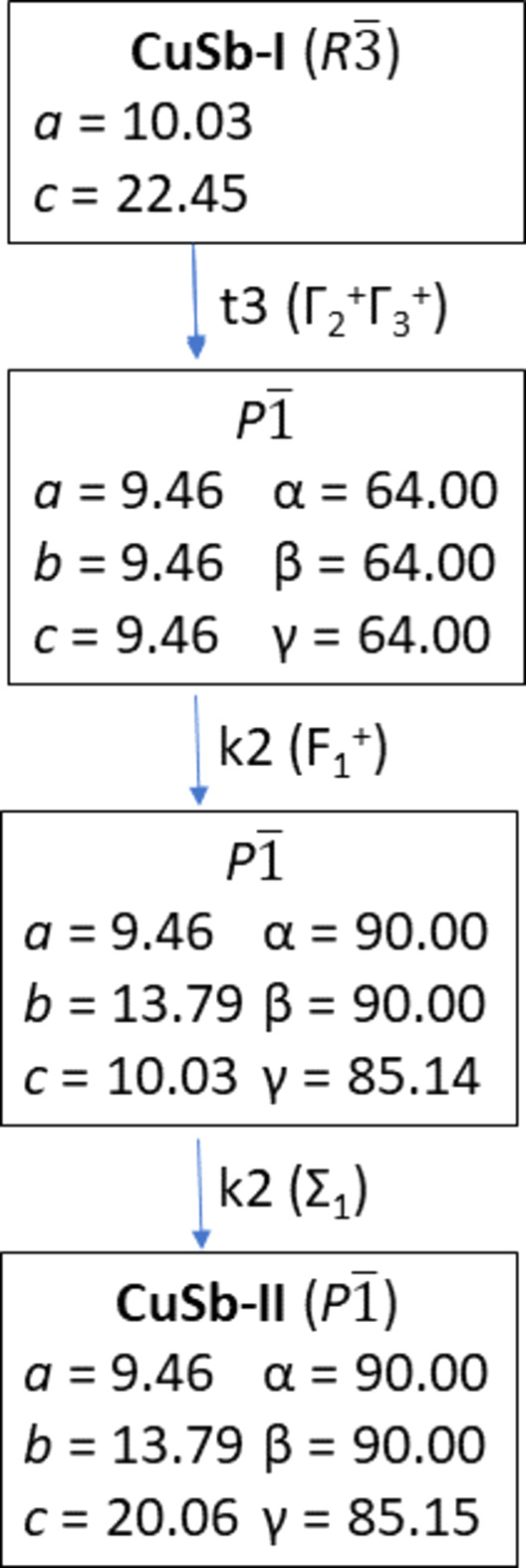
Bärnighausen tree showing the group–subgroup relationship between **CuSb-I** and **CuSb-II**. See Fig. 10 ▸ and text for relationships between basis vectors. The unit-cell dimensions given do not include the effect of strain (see Table 4 ▸).

Inspection of the unit-cell dimensions of **ZnAs-I** (Table 2 ▸) identifies it with the third phase in the Bärnighausen tree of Fig. 4 ▸, the inter­mediate following the F_1_^+^ step in the symmetry pathway leading from ***M*****Sb-I** to ***M*****Sb-II** (***M*** = Cu, Zn). As would be anti­cipated from the discussion in Section 3.1.2, the basis vectors of the unit cells of **ZnSb-II** and **ZnAs-I** are related such that the *c* axis length of **ZnAs-I** is approximately half of that found for **ZnSb-II**, whilst the *a* and *b* axes are similar.

In order to identify the differences between the com­plete structures of **ZnSb-II** and **ZnAs-I**, the structure of the former was transformed using the matrix (100, 010, 00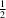) and atoms located within 0.8 Å were merged. The asymmetric unit of the transformed model is shown in blue in Fig. 7 ▸ and we shall refer to it as **T-ZnSb-II**.

Fig. 7 ▸ shows the overlay of the structures of **T-ZnSb-II** and **ZnAs-I**, demonstrating the relationship between them. The Zn and Sb/As positions in the two structures are the same. Zn1 in **ZnAs-I** derives from Zn1 in **ZnSb-II** and Zn2 derives from the superposition of Zn2 and Zn3 from **ZnSb-II**. The orientation of the cations centred on Zn2 in **ZnAs-I** is the same as those centred on Zn3 in **ZnSb-II**. At Zn1, one-third of the XeF_2_ ligands overlay well; the positions of two-thirds of the ligating F atoms are in slightly different positions in the two phases, but the positions of the terminal F atoms are nevertheless similar. As1 sits close to the merged Sb1/Sb3 site of **ZnSb-II**, while the distinct Sb2/4 sites resolve into a single distinct orientation in **ZnAs-I**.

Therefore, the **ZnAs-I** structure can be considered as a modification of the half-*c*-axis averaged cell of **ZnSb-II**. The Zn1 site has rearranged ligand orientations in **ZnAs-I** and Zn2 is the result of adoption of the Zn3 site in **ZnSb-II**. Both anions have re-orientated, with the disorder in Sb1 being resolved in As1, and the anion based on As2 being a rotated form of that of Sb2. A simplified animation of the transition is shown in the file Movie_2 in the supporting information.

The difference in ion orientation between the systems changes the pattern of Xe⋯F contacts, shown projected onto the (2
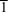
0) planes in Figs. 3 ▸(*e*)–(*f*). The structure contains ap­proximately perpendicular contacts to Xe31, Xe12, Xe22 and Xe32. This results in a total of ten pseudo-equatorial contacts, across two centres, for an average of five per centre. This is similar to the 4.3 per centre seen in **ZnSb-II**, and significantly fewer than the 12 seen in **ZnSb-I**.

#### 3.4.2. ZnAs-II

**ZnAs-I** did not exhibit any structural phase transitions between 100 and 200 K, and pressure was trialled as an additional thermodynamic variable to manipulate the structural behaviour.

A crystal of **ZnAs**, taken from the same sample as that used for the structure analysis of **ZnAs-I**, was exposed to a pressure of 0.15 (5) GPa. The structure belonged to the space group *P*2/*n*, with *Z* = 4 and *Z*′ = 1 (**ZnAs-II**). The question of whether this phase is the result of a transition or a rare ambient-pressure polymorph is difficult to answer definitively. We have not observed this phase for **ZnAs** at ambient pressure across a sampling of 11 crystals. It is generally good practice to confirm the identity of a phase before pressure is applied, but this was not possible in the case of **ZnAs** because of its extreme sensitivity to moisture in the air. While crystals can be mounted on a fibre from under oil and stabilized at low tem­per­a­ture, they decom­pose rapidly on removal from the cold nitro­gen flow of the low-tem­per­a­ture device. Screening of crystals in a closed carefully dried DAC in the absence of a pressure-transmitting medium was also unsuccessful as crystals always decom­posed under these conditions. Data were only collected at one pressure [0.15 (5) GPa], as the sample lost crystallinity after prolonged exposure to the medium.

**ZnAs-II** also possesses the CdCl_2_ motif, with layers shown in Fig. 2 ▸(*d*) and the Xe⋯F anion contacts shown in Figs. 3 ▸(*g*)–(*h*). There are two cations and four anions in the asymmetric unit. Atoms are labelled in the same manner as previously, with suffixes 1 and 2 being bonded to Zn1 and Zn2, respectively, and F atoms with the suffixes 3, 4, 5 and 6 being bonded to As1, As2, As3 and As4. The Zn atoms lie on inversion centres. The Zn—F bond distances are between 1.966 (9) and 2.020 (12) Å. All the [AsF_6_]^−^ anions reside on .2. special positions.

*P*2/*n* is not a subgroup of *R*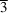 and so **ZnAs-II** does not form part of the Bärnighausen tree shown in Fig. 4 ▸. Nevertheless, for the purposes of visualization of the relationship between the structure and **ZnAs-II**, the space group symmetry of the latter was artificially lowered to *P*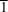 and symmetry mode analysis carried out using *ISODISTORT*. An animation of the transition is shown in Movie_3 in the supporting information. By-and-large, similar comments apply to the relationship between these phases, as was the case between **ZnSb-I** and **ZnAs-I**, in that one set of cations and anions undergo more exaggerated tilting than the remainder. The difference is that an F_1_^+^ mode now causes a pattern of alternating tilts which breaks the translational symmetry of the parent phase, doubling the lengths of the *a* and *b* axes but leaving the space group type unchanged as *R*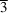. A Γ_2_^+^Γ_3_^+^ step converts the enlarged *R*-cell to its primitive setting with loss of the trigonal symmetry to give the model of **ZnAs-II**.

The above analysis shows that the basis vectors of **ZnAs-II** are related to those of **ZnSb-I** by the matrix
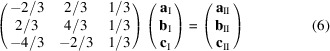


An overlay of the transformed structure of **ZnSb-I** with that of **ZnAs-II** is shown in Fig. 8 ▸. The Zn sites overlap well, with the match extending also to the XeF_2_ ligands for Zn1. The cation based on Zn2 has undergone a reorientation, with each XeF_2_ ligand having an effectively mirrored coordination. The four symmetry-related Sb1 atoms in the transformed cell of **ZnSb-I** sit on the same sites as the four symmetry-distinct As1–As4 sites of **ZnAs-II**. The anions based on As1, As2 and As4 have very similar orientations to those based on Sb1, with only minor tilt changes and distortions. That based on As3 has significantly reoriented, rotating by approximately 45° around the *b* axis.

The relationship between **ZnAs-I** and **ZnAs-II** can be analysed by again lowering the translational symmetry of **ZnAs-I** with the matrix (101/0
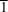
0/10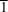), which yields a unit cell of dimensions *a* = 13.355, *b* = 13.209, *c* = 14.188 Å, α = 89.26, β = 97.08, γ = 89.90° and *V* = 2483.5 Å^3^, which are similar to those of phase II. The movie generated from this analysis is available in the supporting information as Movie_4, which shows that lowering of symmetry during a transition of phase II to I occurs as the anions lose their alignment with the .2. axes of phase II.

**Figure 5:**
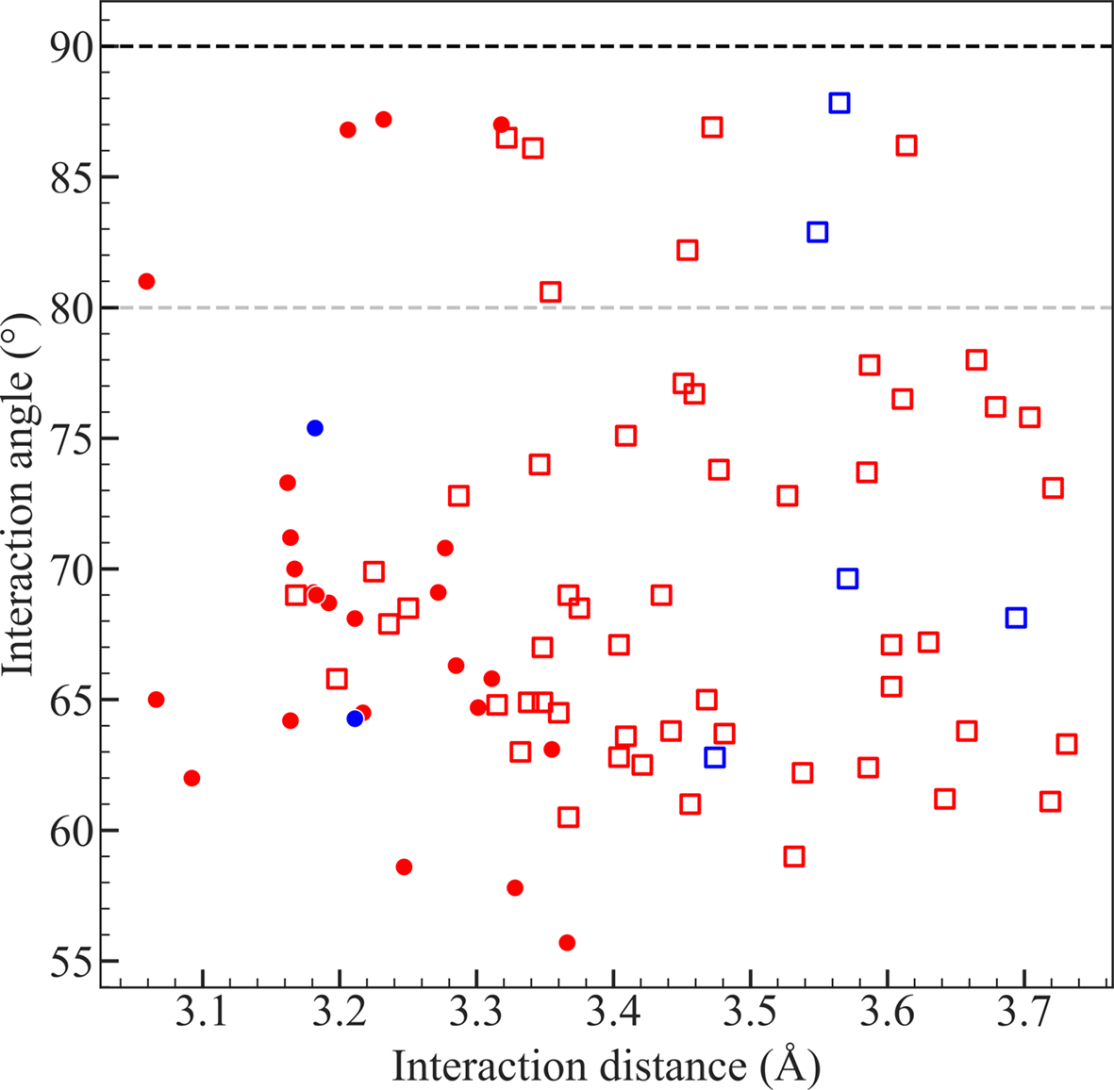
The inter­action angle (∠F⋯Xe—F) of contacts within the sum of the van der Waals radii of F and Xe as a function of inter­action distance for **CuSb-I** and **CuSb-II**. Inter­action angles are shown as the acute angle formed to the F—Xe—F axis. Phase I contacts are shown in blue and phase II contacts are shown in red. Cation–anion contacts are shown as empty squares and cation–cation contacts are shown as filled circles. Pseudo-equatorial contacts, those close to 90°, are shown inside the dashed lines at 80 and 90°. Inter­nal F⋯Xe—F inter­actions formed within the cations have been omitted.

**Figure 6:**
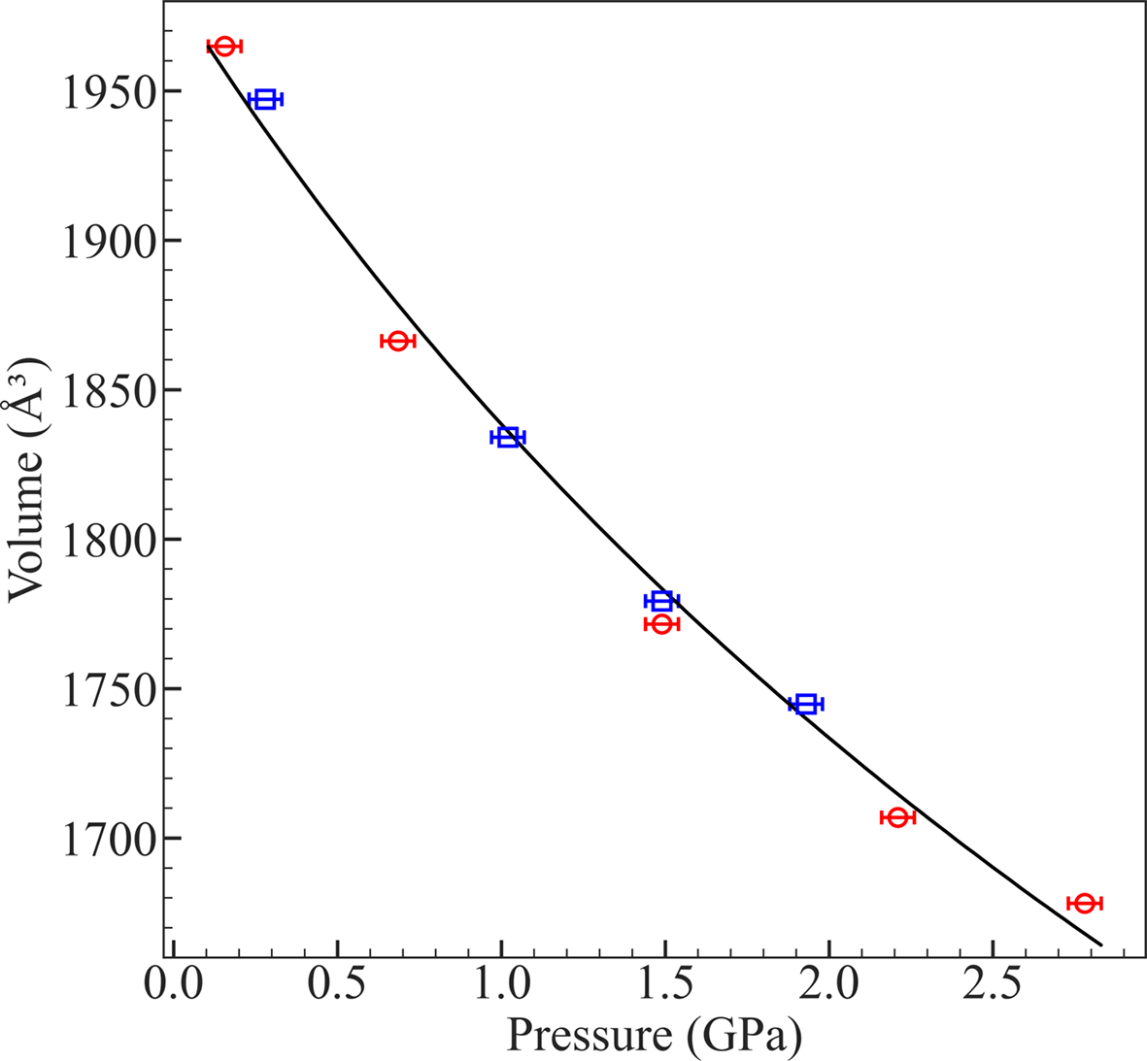
The unit-cell volume as a function of pressure for **CuSb-I** and **ZnSb-I**. Cu data points are given as blue squares and Zn data points are given as red circles. Volume error bars lie fully within the data points but have been omitted for clarity. The trend line shows the second-order Birch–Murnaghan equation of state derived for the combined data sets.

**Figure 7:**
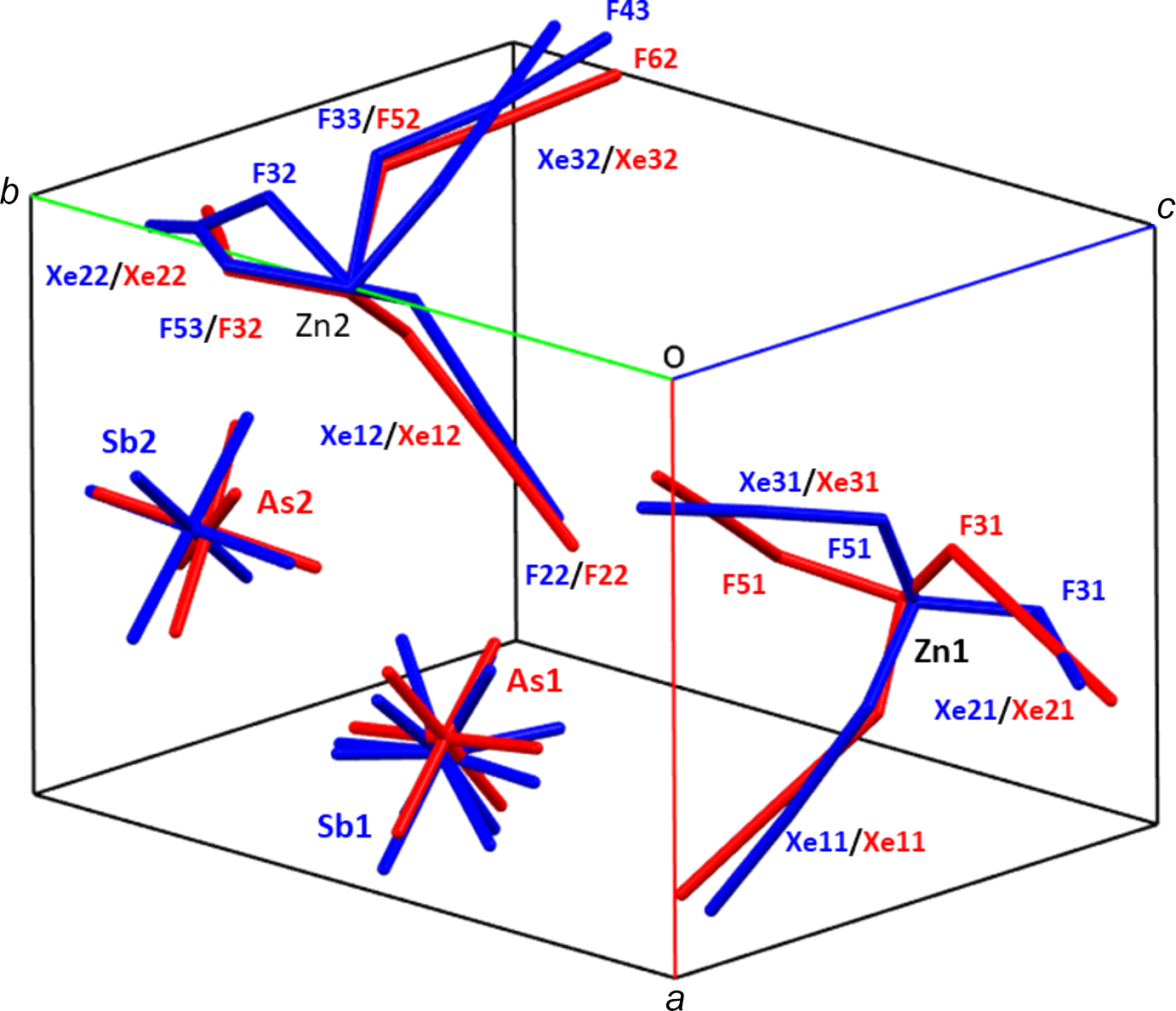
Overlay of the asymmetric units of **T-ZnSb-II** (blue) and **ZnAs-I** (red). Both disorder com­ponents are shown for **T-ZnSb-II**.

**Figure 8:**
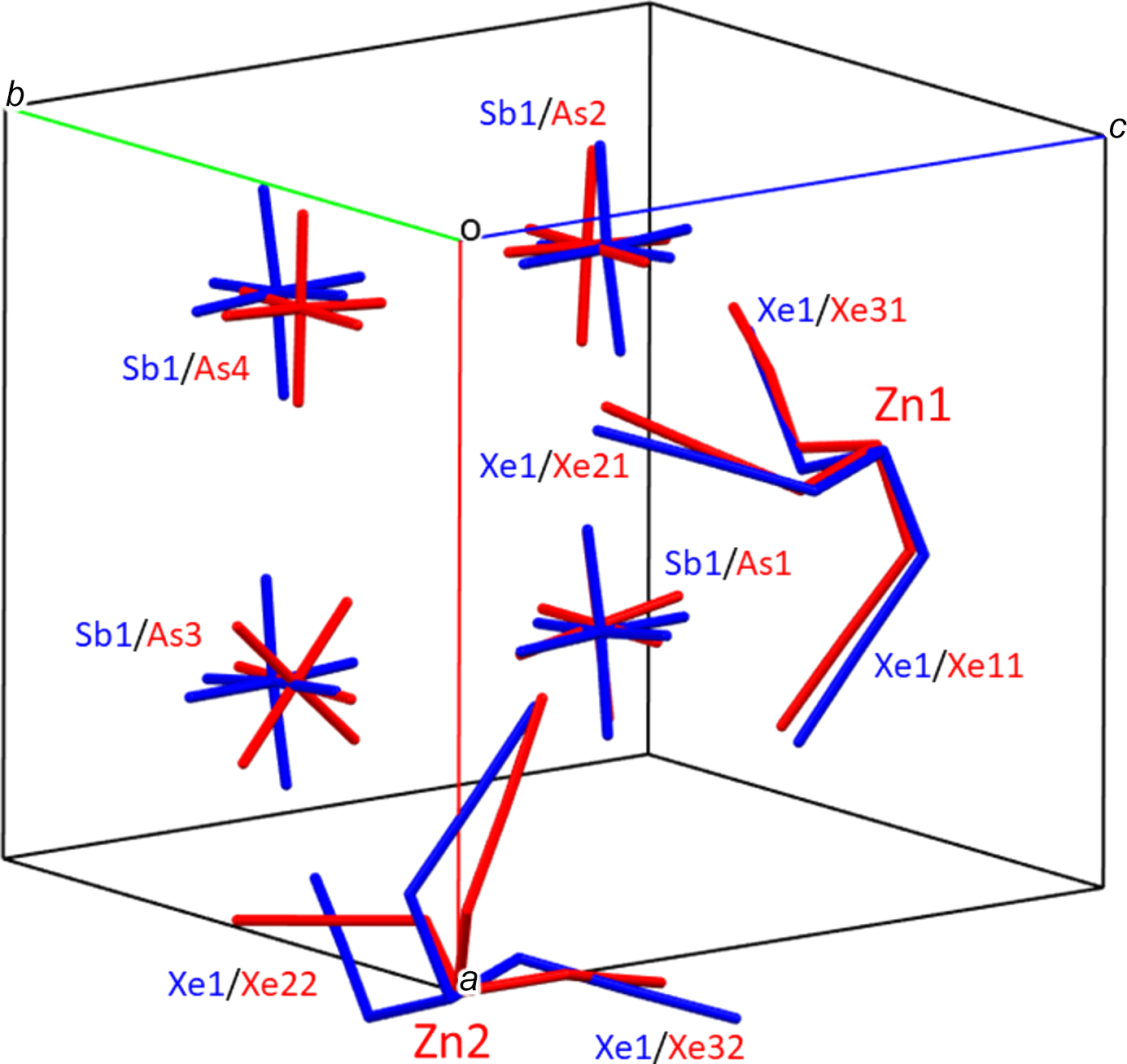
Overlay of **ZnSb-I** (blue) and **ZnAs-II** (red).

**Figure 9:**
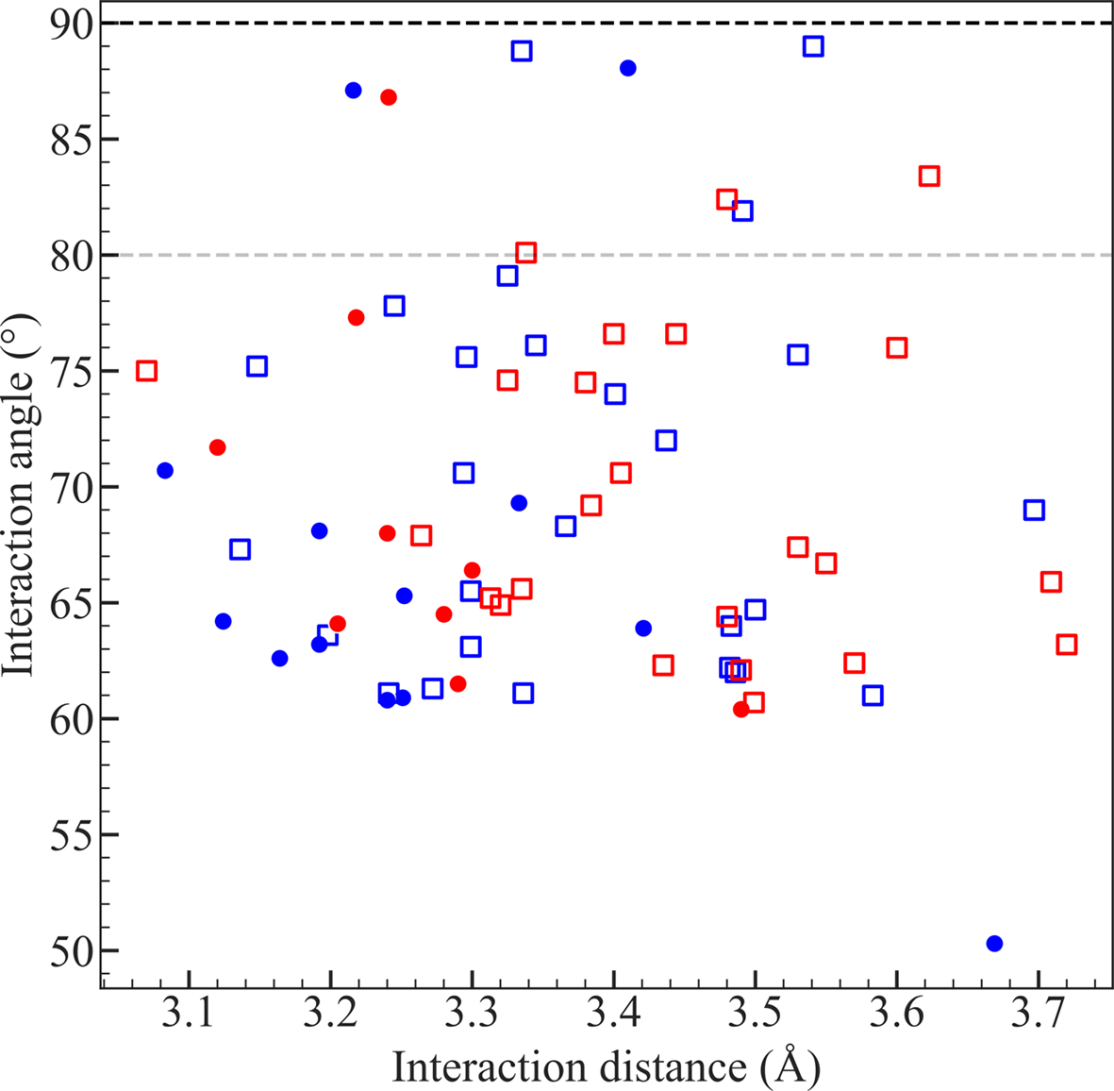
The inter­action angle (∠F⋯Xe—F) of contacts within the sum of the van der Waals radii of F and Xe as a function of inter­action distance for **ZnAs-I** and **ZnAs-II**. Inter­action angles are shown as the acute angle formed to the F—Xe—F axis. Phase I contacts are shown in blue and phase II contacts are shown in red. Cation–anion contacts are shown as squares and cation–cation contacts are shown as circles. Pseudo-equatorial contacts, those close to 90°, are shown inside the dashed lines at 80 and 90°. Inter­actions formed within the cations have been omitted.

Cation–anion contacts are shown in Figs. 3 ▸(*g*)–(*h*). Fig. 9 ▸ shows a plot of contact angle against distance in a similar manner to Fig. 5 ▸ for **ZnAs-I** (blue) and **ZnAs-II** (red). The majority of F⋯Xe—F angles formed by contacts from both anions (squares) and cations (filled circles) fall into the optimal 60–75° range. Cation–cation contacts tend to be shorter than cation–anion contacts, though there is significant overlap, and the inter­action angles are broadly the same. The shortest inter­molecular contact [between Xe22 and F46 at 3.08 (2) Å] occurs between a cation and an anion. There are 10 pseudo-equatorial contacts to F atoms across the two Zn centres, giving an average of five per centre. This is similar to the situation found for **ZnSb-II**.

## 4. Conclusions

The aim of this work was to resolve the disordered Jahn–Teller distortion about the Cu centre in **CuSb-I** by changing the tem­per­a­ture and the applied pressure. Ordering was found to occur on reducing the tem­per­a­ture to 170 K, leading to a change in the space group from *R*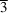 to *P*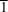 and the formation of a new twinned phase, **CuSb-II**. The transition is displacive, leading to a more com­plex structure in which cations and anions are rotated and the XeF_2_ ligands reoriented relative to the original phase. Symmetry mode analysis shows that the com­plexity of the transition can be broken down into three elemental distortions, which aids the inter­pretation of the transition. The shortest Xe⋯F contacts in **CuSb-I** were formed not between cations and the nearest-neighbour anions, but instead between cations. The transition leads to a marked shortening of the cation–anion contacts and a more general adoption of a contact geometry in which the F⋯Xe—F contact angle lies between 60 and 75°, a range identified as optimal for this class of inter­action (Gomila & Frontera, 2020 ▸).

As our recent work on the actinide com­plexes *M*(O*R*)_4_, where *M* = Th, U or Np and *R* = mesityl, has shown (Shephard *et al.*, 2022 ▸), parallel experiments on isostructural analogues can help to pinpoint the chemical driving forces for phase transitions. In the case of the materials studied here, the same transition as had been observed in **CuSb** was observed in the analogous Zn system **ZnSb**, which demonstrates that the transition is not a consequence of the onset of Jahn–Teller ordering, but is instead associated with the formation of more optimal inter­molecular inter­actions.

Both **CuSb-I** and **ZnSb-I** were shown to be stable to pressure, reaching a maximum of 2.78 (5) GPa for the Zn system.

We have, as part of this work, also determined the crystal structures of the [AsF_6_]^−^-containing system **ZnAs**. This is somewhat less stable than the Sb-containing systems, and decom­poses slowly at room tem­per­a­ture. At low tem­per­a­ture, the structure of **ZnAs** is triclinic, but it appears to become monoclinic even at the very modest pressure of 0.15 GPa. Both structures are closely related to those of the Sb analogues **ZnAs-I** as an inter­mediate in the pathway observed in the Sb-containing analogues, and **ZnAs-II** through symmetry loss in an enlarged rhombohedral phase. Indeed, all the structures reported here can be inter­preted in terms of distortions of a CdCl_2_ aristotype.

**Figure 10:**
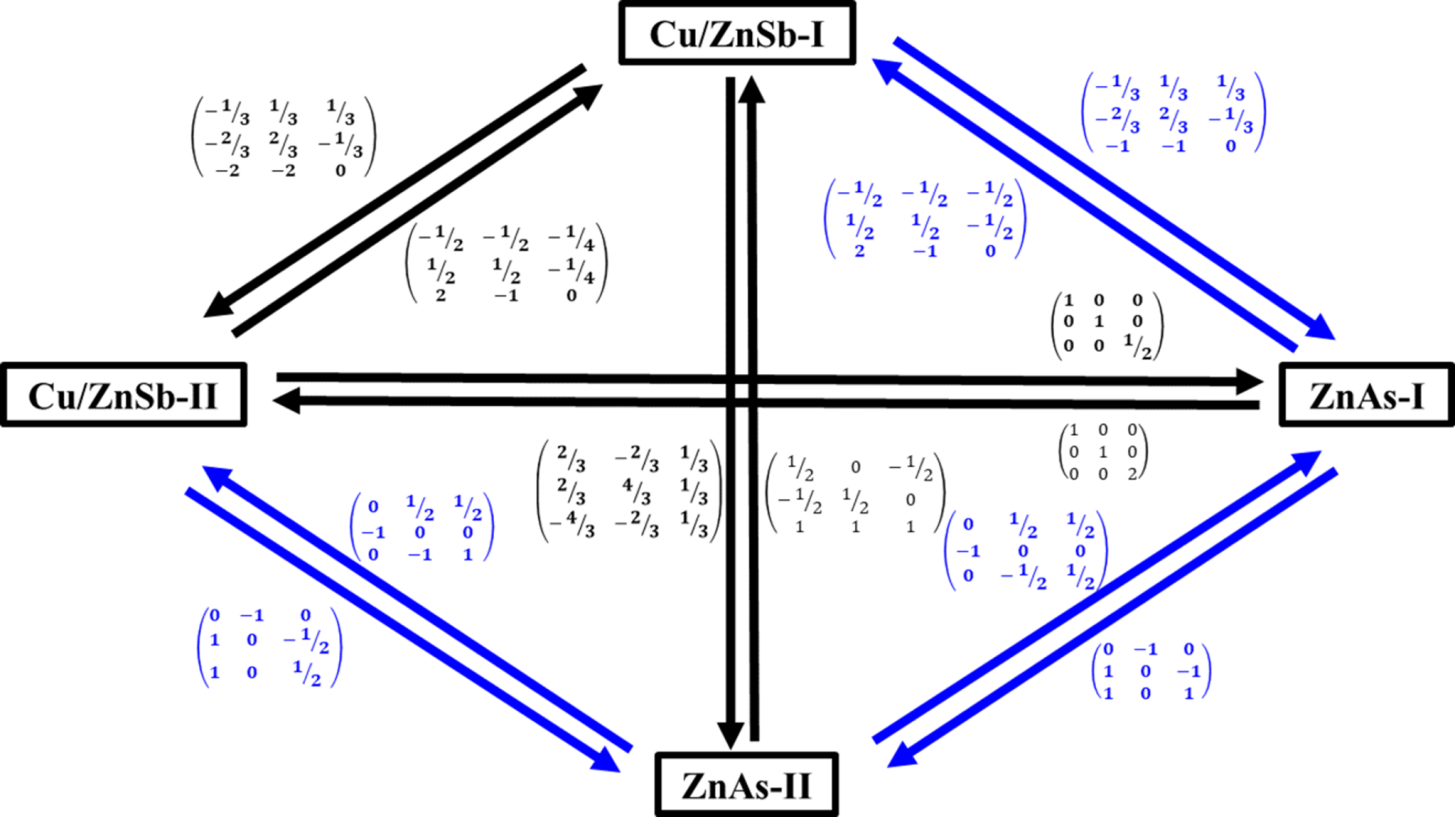
A summary of the transformation matrices relating basis vectors of phases described in this article. The transitions discussed in the text are shown in black, while those derived by combination of other matrices are shown in blue.

The relationships between the basis vectors of the different phases could be determined directly from the orientation matrices of the different phases if the transition was observed *in situ*, or from the symmetry mode analysis. These relationships, which are summarized in Fig. 10 ▸, enable key differences between phases to be identified by superposition of two phases in a common set of axes. The unit-cell dimensions ob­tained by application of the transformations shown in Fig. 10 ▸, starting from those of **ZnSb-I** at 200 K, are com­pared with the observed dimensions for each phase in Table 4 ▸; com­parison of pairs of dimensions provides a measure of the strain generated over the course of a transition.

## Supplementary Material

Crystal structure: contains datablock(s) CuSbI_200K, CuSbII_170K, CuSbII_30K, cusbi_0p28gpa, cusbi_1p03gpa, cusbi_1p49gpa, cusbi_1p93gpa, ZnSbI_200K, znsbi_0p16gpa, znsbi_0p69gpa, znsbi_1p49gpa, znsbi_2p21gpa, znsbi_2p78gpa, ZnAsI_100K, ZnAsII_0p15GPa, global, ZnSbII_160K. DOI: 10.1107/S2052252526003751/yc5054sup1.cif

Structure factors: contains datablock(s) CuSbI_200K. DOI: 10.1107/S2052252526003751/yc5054CuSbI_200Ksup2.hkl

Structure factors: contains datablock(s) CuSbII_170K. DOI: 10.1107/S2052252526003751/yc5054CuSbII_170Ksup3.hkl

Structure factors: contains datablock(s) CuSbII_30K. DOI: 10.1107/S2052252526003751/yc5054CuSbII_30Ksup4.hkl

Structure factors: contains datablock(s) cusbi_0p28gpa. DOI: 10.1107/S2052252526003751/yc5054cusbi_0p28gpasup5.hkl

Structure factors: contains datablock(s) cusbi_1p03gpa. DOI: 10.1107/S2052252526003751/yc5054cusbi_1p03gpasup6.hkl

Structure factors: contains datablock(s) cusbi_1p49gpa. DOI: 10.1107/S2052252526003751/yc5054cusbi_1p49gpasup7.hkl

Structure factors: contains datablock(s) cusbi_1p93gpa. DOI: 10.1107/S2052252526003751/yc5054cusbi_1p93gpasup8.hkl

Structure factors: contains datablock(s) ZnSbI_200K. DOI: 10.1107/S2052252526003751/yc5054ZnSbI_200Ksup9.hkl

Structure factors: contains datablock(s) znsbi_0p16gpa. DOI: 10.1107/S2052252526003751/yc5054znsbi_0p16gpasup10.hkl

Structure factors: contains datablock(s) znsbi_0p69gpa. DOI: 10.1107/S2052252526003751/yc5054znsbi_0p69gpasup11.hkl

Structure factors: contains datablock(s) znsbi_1p49gpa. DOI: 10.1107/S2052252526003751/yc5054znsbi_1p49gpasup12.hkl

Structure factors: contains datablock(s) znsbi_2p21gpa. DOI: 10.1107/S2052252526003751/yc5054znsbi_2p21gpasup13.hkl

Structure factors: contains datablock(s) znsbi_2p78gpa. DOI: 10.1107/S2052252526003751/yc5054znsbi_2p78gpasup14.hkl

Structure factors: contains datablock(s) ZnAsI_100K. DOI: 10.1107/S2052252526003751/yc5054ZnAsI_100Ksup15.hkl

Structure factors: contains datablock(s) ZnAsII_0p15GPa. DOI: 10.1107/S2052252526003751/yc5054ZnAsII_0p15GPasup16.hkl

Structure factors: contains datablock(s) ZnSbII_160K. DOI: 10.1107/S2052252526003751/yc5054ZnSbII_160Ksup17.hkl

Additional figures and tables. DOI: 10.1107/S2052252526003751/yc5054sup18.pdf

Movie_1. DOI: 10.1107/S2052252526003751/yc5054sup19.gif

Movie_2. DOI: 10.1107/S2052252526003751/yc5054sup20.gif

Movie_3. DOI: 10.1107/S2052252526003751/yc5054sup21.gif

Movie_4. DOI: 10.1107/S2052252526003751/yc5054sup22.gif

CCDC references: 2545043, 2545042, 2545041, 2545040, 2545039, 2545038, 2545037, 2545036, 2545035, 2545034, 2545033, 2545032, 2545031, 2545030, 2545029, 2545028

## Figures and Tables

**Table 1:** Crystal and refinement data for **CuSb** and **ZnSb** in phases I and II

Chemical formula	[Cu(XeF_2_)_6_][SbF_6_]_2_ (**CuSb-I**)	[Cu(XeF_2_)_6_][SbF_6_]_2_ (**CuSb-II)**	[Zn(XeF_2_)_6_][SbF_6_]_2_ (**ZnSb-I**)	[Zn(XeF_2_)_6_][SbF_6_]_2_ (**ZnSb-II**)
Phase	I	II	I	II
Temperature (K)	200	170	200	160
*M*_r_	1550.84	1550.84	1552.67	1552.63
Crystal system, space group	Trigonal, *R* 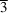	Triclinic, *P* 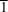	Trigonal, *R* 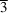	Triclinic, *P* 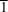
*a*, *b*, *c* (Å)	10.0302 (3), 10.0302 (3), 22.4539 (9)	9.4864 (13), 13.7227 (17), 19.880 (3)	10.0781 (4), 10.0781 (4), 22.4417 (13)	9.4869 (10), 13.7005 (16), 20.057 (2)
α, β, γ (°)	90, 90, 120	89.503 (4), 88.661 (4), 87.490 (4)	90, 90, 120	89.469 (3), 88.697 (3), 86.951 (3)
*V* (Å^3^)	1956.33 (13)	2584.7 (6)	1974.0 (2)	2602.4 (5)
*Z*	3	4	3	4
Radiation type	Ag *K*α, λ = 0.56086 Å	Ag *K*α, λ = 0.56086 Å	Ag *K*α, λ = 0.56086 Å	Ag *K*α, λ = 0.56086 Å
μ (mm^−1^)	5.57	5.62	5.65	5.63
Crystal size (mm)	0.40 × 0.30 × 0.10	0.40 × 0.30 × 0.10	0.30 × 0.25 × 0.20	0.30 × 0.25 × 0.20
				
Data collection				
No. of measured, independent and observed [*I* > 2σ(*I*)] reflections	16946, 898, 871	51710, 10437, 9427	19496, 1358, 1293	56066, 10213, 7491
*R*_int_	0.042	0.0453	0.067	0.089
(sin θ/λ)_max_ (Å^−1^)	0.626	0.625	0.624	0.627
				
Refinement				
*R*[*F*^2^ > 2σ(*F*^2^)], *wR*(*F*^2^), *S*	0.015, 0.036, 1.08	0.041, 0.117, 1.08	0.033, 0.091, 1.19	0.061, 0.094, 1.03
No. of reflections	898	10437	1358	9888
No. of parameters	52	599	52	598
No. of restraints	18	423	18	0
Δρ_max_, Δρ_min_ (e Å^−3^)	0.62, −0.47	4.15, −1.82	0.75, −1.15	3.45, −2.48

**Table 2:** Crystal and refinement data for **ZnAs-I** and **ZnAs-II**

Chemical formula	[Zn(XeF_2_)_6_][AsF_6_]_2_ (**ZnAs-I**)	[Zn(XeF_2_)_6_][AsF_6_]_2_ at 0.15 GPa (**ZnAs-II**)
Phase	I	II
Temperature (K)	100	298
*M*_r_	1459.01	1459.01
Crystal system, space group	Triclinic, *P* 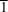	Monoclinic, *P*2/*n*
*a*, *b*, *c* (Å)	9.1236 (7), 13.2086 (10), 10.3242 (9)	13.630 (2), 13.6939 (9), 14.1449 (8)
α, β, γ (°)	89.555 (3), 93.491 (3), 90.650 (3)	90, 90.497 (10), 90
*V* (Å^3^)	1241.75 (17)	2640.0 (4)
*Z*	2	4
Radiation type	Mo *K*α λ = 0.71073 Å	Synchrotron, λ = 0.1582 Å
μ (mm^−1^)	11.85	0.83
Crystal size (mm)	0.40 × 0.20 × 0.20	0.25 × 0.22 × 0.05
		
Data Collection		
No. of measured, independent and observed [*I* > 2σ(*I*)] reflections	42983, 8946, 8140	5517, 2927, 2267
*R*_int_	0.089	0.036
(sin θ/λ)_max_ (Å^−1^)	0.625	0.625
		
Refinement		
*R*[*F*^2^ > 2σ(*F*^2^)], *wR*(*F*^2^), *S*	0.052, 0.148, 1.17	0.062, 0.197, 1.08
No. of reflections	8946	2927
No. of parameters	304	307
No. of restraints	198	477
Δρ_max_, Δρ_min_ (e Å^−3^)	2.13, −1.79	1.77, −1.18

**Table 3:** Crystal and refinement data for **CuSb** and **ZnSb** at selected pressures

Chemical formula	[Cu(XeF_2_)_6_][SbF_6_]_2_ at 0.28 GPa	[Cu(XeF_2_)_6_][SbF_6_]_2_ at 1.93 GPa	[Zn(XeF_2_)_6_][SbF_6_]_2_ at 0.16 GPa	[Zn(XeF_2_)_6_][SbF_6_]_2_ at 2.78 GPa
Phase	I	I	I	I
*M*_r_	1550.84	1550.84	1552.67	1552.67
Crystal system, space group	Trigonal, *R* 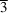	Trigonal, *R* 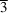	Trigonal, *R* 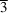	Trigonal, *R* 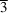
Temperature (K)	298	298	298	298
*a*, *b*, *c* (Å)	10.0084 (4), 10.0084 (4), 22.4492 (7)	9.6632 (7), 9.6632 (7), 21.6075 (11)	10.0363 (4), 10.0363 (4), 22.5388 (11)	9.5373 (10), 9.5373 (10), 21.292 (2)
α, β, γ (°)	90, 90, 120	90, 90, 120	90, 90, 120	90, 90, 120
*V* (Å^3^)	1947.43 (17)	1747.34 (19)	1966.11 (18)	1677.3 (4)
*Z*	3	3	3	3
Radiation type	Synchrotron, λ = 0.2906 Å	Synchrotron, λ = 0.2906 Å	Synchrotron, λ = 0.2906 Å	Synchrotron, λ = 0.2906 Å
μ (mm^−1^)	5.24	5.60	4.99	5.84
Crystal size (mm)	0.20 × 0.10 × 0.05	0.20 × 0.10 × 0.05	0.30 × 0.25 × 0.20	0.30 × 0.25 × 0.20
				
Data collection				
No. of measured, independent and observed [*I* > 2σ(*I*)] reflections	1503, 692, 679	1185, 587, 548	1559, 808, 747	1179, 624, 407
*R*_int_	0.030	0.017	0.022	0.031
(sin θ/λ)_max_ (Å^−1^)	0.624	0.624	0.625	0.624
				
Refinement				
*R*[*F*^2^ > 2σ(*F*^2^)], *wR*(*F*^2^), *S*	0.058, 0.137, 1.17	0.060, 0.169, 1.16	0.059, 0.171, 1.10	0.081, 0.266, 1.06
No. of reflections	692	587	808	624
No. of parameters	52	51	52	51
No. of restraints	18	18	18	18
Δρ_max_, Δρ_min_(e Å^−3^)	1.32, −2.09	1.29, −1.15	0.98, −1.51	2.50, −1.44

**Table 4:** Comparison of the observed unit-cell dimensions for phases of **Zn*A*** (***A*** = As, Sb) with ideal strain-free values calculated from the underlying relationships between the basis vectors shown in Fig. 10 ▸

Phase	Space group		*a* (Å)	*b* (Å)	*c* (Å)	α (°)	β (°)	γ (°)	*V* (Å^3^)	*T* (K)	*P* (GPa)
**ZnSb-I**	*R* 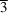	obs.	10.078	10.078	22.442	90	90	120	1974.0	200	0
**ZnSb-II**	*P* 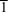	ideal	9.476	13.834	20.156	90	90	84.85	2631.7		
		obs.	9.482	13.705	20.048	89.51	88.72	86.96	2600.7	160	0
**ZnAs-I**	*P* 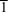	ideal	9.476	13.834	10.078	90	90	84.85	1315.9		
		obs.	9.124	13.209	10.324	89.56	93.49	90.65	1241.8	100	0
**ZnAs-II**	*P*2/*n*	ideal	13.834	13.833	13.833	93.53	93.52	93.52	2631.7		
		obs.	13.63	13.694	14.145	90	90.5	90	2640.0	298	0.15

## References

[bb1] Alvarez, S., Avnir, D., Llunell, M. & Pinsky, M. (2002). *New J. Chem.* **26**, 996–1009.

[bb2] Astley, T., Ellis, P. J., Freeman, H. C., Hitchman, M. A., Keene, F. R. & Tiekink, E. R. T. (1995). *J. Chem. Soc. Dalton Trans.* pp. 595–601.

[bb3] Bartlett, N. (1962). *Proc. Chem. Soc.* **6**, 218.

[bb4] Beddard, G. S., Halcrow, M. A., Hitchman, M. A., de Miranda, M. P., Simmons, C. J. & Stratemeier, H. (2003). *Dalton Trans.* pp. 1028–1032.

[bb5] Bersuker, I. B. (2021). *Chem. Rev.* **121**, 1463–1512. 10.1021/acs.chemrev.0c00718 33353296

[bb6] Betteridge, P. W., Carruthers, J. R., Cooper, R. I., Prout, K. & Watkin, D. J. (2003). *J. Appl. Cryst.* **36**, 1487–1487.

[bb7] Birch, F. (1947). *Phys. Rev.* **71**, 809–824.

[bb8] Blessing, R. H. (1995). *Acta Cryst.* A**51**, 33–38. 10.1107/s0108767394005726 7702794

[bb9] Boehler, R. & De Hantsetters, K. (2004). *High Pressure Res.* **24**, 391–396.

[bb10] Brock, D. S., Schrobilgen, G. J. & Žemva, B. (2013). *Comprehensive Inorganic Chemistry II*, edited by J. Reedijk & K. Poeppelmeier, pp. 755–822. Oxford: Elsevier.

[bb11] Bruker (2024). *APEX5* and *SAINT*. Bruker AXS Inc., Madison, Wisconsin, USA.

[bb12] Campbell, B. J., Stokes, H. T., Tanner, D. E. & Hatch, D. M. (2006). *J. Appl. Cryst.* **39**, 607–614.

[bb13] Chernick, C. L., Claassen, H. H., Fields, P. R., Hyman, H. H., Malm, J. G., Manning, W. M., Matheson, M. S., Quarterman, L. A., Schreiner, F., Selig, H. H., Sheft, I., Siegel, S., Sloth, E. N., Stein, L., Studier, M. H., Weeks, J. L. & Zirin, M. H. (1962). *Science* **138**, 136–138. 10.1126/science.138.3537.136 17818399

[bb14] Cirera, J., Ruiz, E. & Alvarez, S. (2005). *Organometallics* **24**, 1556–1562.

[bb15] Claassen, H. H., Selig, H. & Malm, J. G. (1962). *J. Am. Chem. Soc.* **84**, 3593–3593.

[bb16] De Backere, J. R. & Schrobilgen, G. J. (2018). *Angew. Chem. Int. Ed.* **57**, 13167–13171. 10.1002/anie.201807755 30091818

[bb17] Deeth, R. J. & Hitchman, M. A. (1986). *Inorg. Chem.* **25**, 1225–1233.

[bb18] Dolomanov, O. V., Bourhis, L. J., Gildea, R. J., Howard, J. A. K. & Puschmann, H. (2009). *J. Appl. Cryst.* **42**, 339–341.

[bb19] Francis, E. A., Scharinger, S., Németh, K., Kamarás, K. & Kuntscher, C. A. (2012). *Phys. Rev. B* **85**, 195428.

[bb20] Funnell, N. P., Dawson, A., Francis, D., Lennie, A. R., Marshall, W. G., Moggach, S. A., Warren, J. E. & Parsons, S. (2010). *CrystEngComm* **12**, 2573–2583.

[bb21] Gaazo, J. (1976). *Coord. Chem. Rev.* **19**, 253–297.

[bb22] Gantar, D., Leban, I., Frlec, B. & Holloway, J. H. (1987). *J. Chem. Soc. Dalton Trans.* pp. 2379–2383.

[bb23] Gomila, R. & Frontera, A. (2020). *Front. Chem.* **8**, 395. 10.3389/fchem.2020.00395 PMC7218167 32435634

[bb24] Groom, C. R., Bruno, I. J., Lightfoot, M. P. & Ward, S. C. (2016). *Acta Cryst.* B**72**, 171–179. 10.1107/S2052520616003954 PMC4822653 27048719

[bb25] Halder, G. J., Chapman, K. W., Schlueter, J. A. & Manson, J. L. (2011). *Angew. Chem. Int. Ed.* **50**, 419–421. 10.1002/anie.201003380 20836108

[bb26] Hoppe, R., Dähne, W., Mattauch, H. & Rödder, K. M. (1962). *Angew. Chem.* **74**, 903.

[bb27] Jørgensen, J.-E. & Smith, R. I. (2006). *Acta Cryst.* B**62**, 987–992. 10.1107/S0108768106030023 17108651

[bb28] Kantor, I., Prakapenka, V., Kantor, A., Dera, P., Kurnosov, A., Sinogeikin, S., Dubrovinskaia, N. & Dubrovinsky, L. (2012). *Rev. Sci. Instrum.* **83**, 125102. 10.1063/1.4768541 23278021

[bb29] Kirshenboim, O. & Kozuch, S. (2016). *J. Phys. Chem. A* **120**, 9431–9445. 10.1021/acs.jpca.6b07894 27783513

[bb30] Krause, L., Herbst-Irmer, R., Sheldrick, G. M. & Stalke, D. (2015). *J. Appl. Cryst.* **48**, 3–10. 10.1107/S1600576714022985 PMC4453166 26089746

[bb31] Liermann, H.-P., Konôpková, Z., Morgenroth, W., Glazyrin, K., Bednarčik, J., McBride, E. E., Petitgirard, S., Delitz, J. T., Wendt, M., Bican, Y., Ehnes, A., Schwark, I., Rothkirch, A., Tischer, M., Heuer, J., Schulte-Schrepping, H., Kracht, T. & Franz, H. (2015). *J. Synchrotron Rad.* **22**, 908–924. 10.1107/S1600577515005937 PMC4489534 26134794

[bb32] Lozinšek, M., Mercier, H. P. A. & Schrobilgen, G. J. (2021). *Angew. Chem. Int. Ed.* **60**, 8149–8156. 10.1002/anie.202014682 PMC8048594 33242230

[bb33] Macrae, C. F., Sovago, I., Cottrell, S. J., Galek, P. T. A., McCabe, P., Pidcock, E., Platings, M., Shields, G. P., Stevens, J. S., Towler, M. & Wood, P. A. (2020). *J. Appl. Cryst.* **53**, 226–235. 10.1107/S1600576719014092 PMC6998782 32047413

[bb34] Malm, J. G., Sheft, I. & Chernick, C. L. (1963). *J. Am. Chem. Soc.* **85**, 110–111.

[bb35] Merrill, L. & Bassett, W. A. (1974). *Rev. Sci. Instrum.* **45**, 290–294.

[bb36] Motaln, K., Uran, E., Giordano, N., Parsons, S. & Lozinšek, M. (2025). *J. Appl. Cryst.* **58**, 221–226. 10.1107/S1600576725000342 PMC11798517 39917187

[bb37] Mržljak, T., Goreshnik, E., Tavčar, G. & Tramšek, M. (2025). *Eur. J. Inorg. Chem.* **28**, e202500275.

[bb38] Müller, U. (2013). In *Symmetry Relationships Between Crystal Structures*. Oxford University Press.

[bb39] Novelli, G., Maynard-Casely, H. E., McIntyre, G. J., Warren, M. R. & Parsons, S. (2020). *Cryst. Growth Des.* **20**, 7788–7804.

[bb40] Putz, H. & Brandenberg, K. (2024). *DIAMOND*. Version 5.1. Crystal Impact GbR, Bonn, Germany.

[bb41] Rigaku (2023). *CrysAlis PRO* and *SCALE3 ABSPACK*. Rigaku Corporation, Wrocław, Poland.

[bb42] Sheldrick, G. M. (2015*a*). *Acta Cryst.* A**71**, 3–8.

[bb43] Sheldrick, G. M. (2015*b*). *Acta Cryst.* C**71**, 3–8.

[bb44] Shen, G., Wang, Y., Dewaele, A., Wu, C., Fratanduono, D. E., Eggert, J., Klotz, S., Dziubek, K. F., Loubeyre, P., Fat’yanov, O. V., Asimow, P. D., Mashimo, T. & Wentzcovitch, R. M. M. (2020). *High Pressure Res.* **40**, 299–314.

[bb45] Shephard, J. J., Berryman, V. E. J., Ochiai, T., Walter, O., Price, A. N., Warren, M. R., Arnold, P. L., Kaltsoyannis, N. & Parsons, S. (2022). *Nat. Commun.* **13**, 5923. 10.1038/s41467-022-33459-7 PMC9546877 36207297

[bb47] Slivnik, J., Brčić, B., Volavšek, B., Šmalc, A., Frlec, B., Zemljič, R., Anžur, A. & Veksli, Z. (1962*a*). *Croat. Chem. Acta* **34**, 187–188.

[bb46] Slivnik, J., Brčić, B., Volavšek, B., Marsel, J., Vrščaj, V., Šmalc, A., Frlec, B. & Zemljič, Z. (1962*b*). *Croat. Chem. Acta* **34**, 253.

[bb48] Šmalc, A. & Lutar, K. (1992). *Inorg. Synth.* **29**, 1–4.

[bb49] Spek, A. L. (2020). *Acta Cryst.* E**76**, 1–11. 10.1107/S2056989019016244 PMC6944088 31921444

[bb50] Stokes, H. T., Hatch, D. M. & Campbell, B. J. (2025). *ISODISTORT* in *ISOTROPY Software Suite*. https://iso.byu.edu/.

[bb51] Tavčar, G., Goreshnik, E. & Mazej, Z. (2006). *J. Fluorine Chem.* **127**, 1368–1373.

[bb52] Thomas, S. P., Worthy, A., Eikeland, E. Z., Thompson, A. J., Grosjean, A., Tolborg, K., Krause, L., Sugimoto, K., Spackman, M. A., McMurtrie, J. C., Clegg, J. K. & Iversen, B. B. (2023). *Chem. Mater.* **35**, 2495–2502.

[bb53] Thorn, A., Dittrich, B. & Sheldrick, G. M. (2012). *Acta Cryst.* A**68**, 448–451.

[bb54] Tramšek, M., Benkič, P. & Žemva, B. (2004). *Angew. Chem. Int. Ed.* **43**, 3456–3458. 10.1002/anie.200453802 15221838

[bb55] Tramšek, M. & Žemva, B. (2006). *Acta Chim. Slov.* **53**, 105–116.

[bb56] Vogt, J. & Alvarez, S. (2014). *Inorg. Chem.* **53**, 9260–9266. 10.1021/ic501364h 25144450

[bb57] Weaver, E. E., Weinstock, B. & Knop, C. P. (1963). *J. Am. Chem. Soc.* **85**, 111–112.

[bb58] Weeks, J. L., Chernick, C. L. & Matheson, M. S. (1962). *J. Am. Chem. Soc.* **84**, 4612–4613.

