# Supplementary material for: Phase relationships in homoleptic com­plexes of XeF2
Source: IUCrJ. 2026 May 18;13(Pt 4):420–32. doi: 10.1107/S2052252526003751 (PMC13324652; doi:10.1107/S2052252526003751)
Supplement: Supplementary file 18 [file m-13-00420-sup18.pdf]

IUCrJ

Volume 13 (2026)

Supporting information for article:

Phase relationships in homoleptic complexes of XeF2

Lewis A. Clough, Kristian Radan, Dominik Daisenberger, Joseph Hriljac, Nico Giordano, Matic Lozinšek and Simon Parsons

Phase Relationships in Homoleptic Complexes of XeF_2

Lewis A. Clough,[a,b] Kristian Radan,[c] Dominik Daisenberger,[b] Joseph Hriljac,[b] Nico Giordano,[d] Matic Lozinšek[c]* and Simon Parsons[a]*

[a] EaStCHEM School of Chemistry and Centre for Science at Extreme Conditions, University of Edinburgh, King's Buildings, West Mains Road, Edinburgh, EH9 3FJ, United Kingdom

[b] Diamond Light Source, Harwell Science and Innovation Campus, Didcot, Oxfordshire, OX11 0DE, UK

[c] Extreme Conditions Chemistry Laboratory, Jožef Stefan Institute, Jamova cesta 39, 1000 Ljubljana, Slovenia

[d] Deutsches Elektronen-Synchrotron DESY, 22607 Hamburg, Germany

Supplementary material

Contents

Figure S1. Synthetic apparatus 2

Figure S2. The Temperature-Volume relationship in **CuSb** and **ZnSb** 3

Figure S3. Crystal packing in $CdCl_2$ 4

Table S1. The unit cell volume of **CuSb** as a function of temperature 5

Table S2. The unit cell volume of **ZnSb** as a function of temperature 6

Table S3. Distortion analysis of the metal sites in all phases relative to **MSb-I** (**M**=Cu or Zn) 6

Table S4. The unit cell volume of **ZnAs-I** as a function of temperature 6

Movie S1. Animation of the **CuSb-I** to **CuSb-II** transformation (separate file)

Movie S2. Animation of the **ZnSb-I** to **ZnAs-I** transformation (separate file)

Movie S3. Animation of the **ZnSb-I** to **ZnAs-II** transformation (separate file)

Movie S4. Animation of the **ZnAs-I** to **ZnAs-II** transformation (separate file)

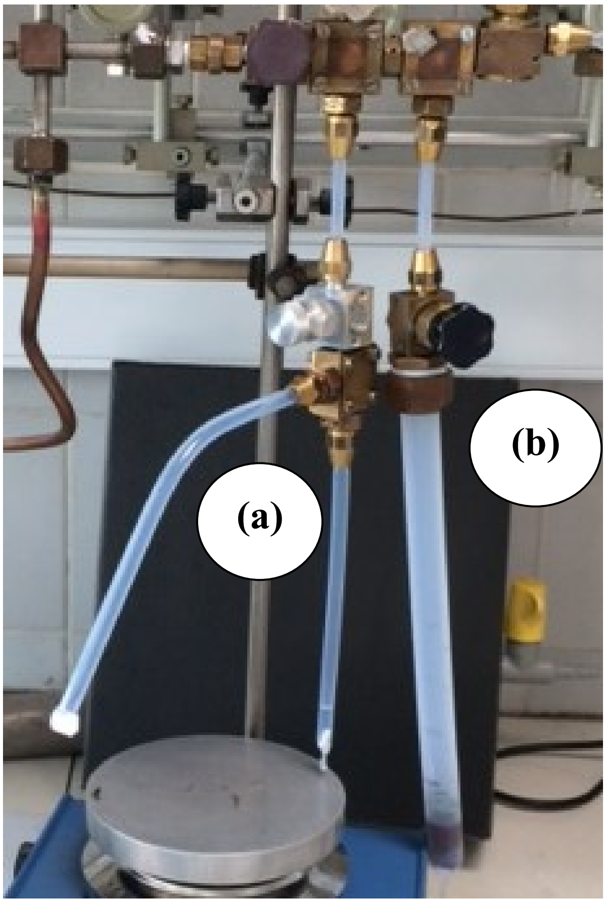

Figure S1. Showing the reaction vessel of the type used in the synthesis of all compounds in this work at (a). Anhydrous HF with dissolved $K_2Ni^{IV}F_6$ is shown at (b).

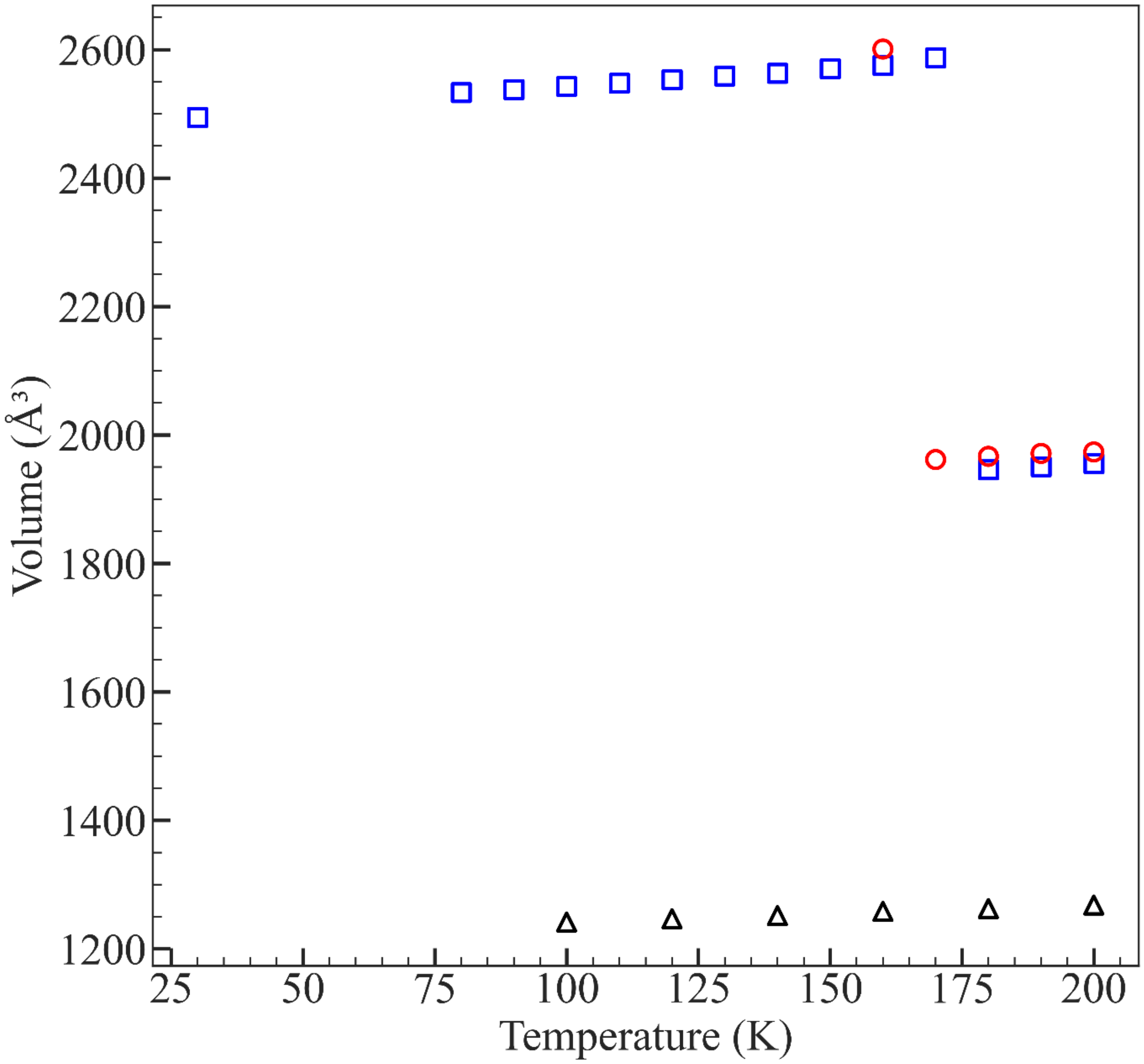

Figure S2. A plot of the unit cell volume against temperature for **CuSb** (blue squares), **ZnSb** (red circles) and **ZnAs** (black triangles).

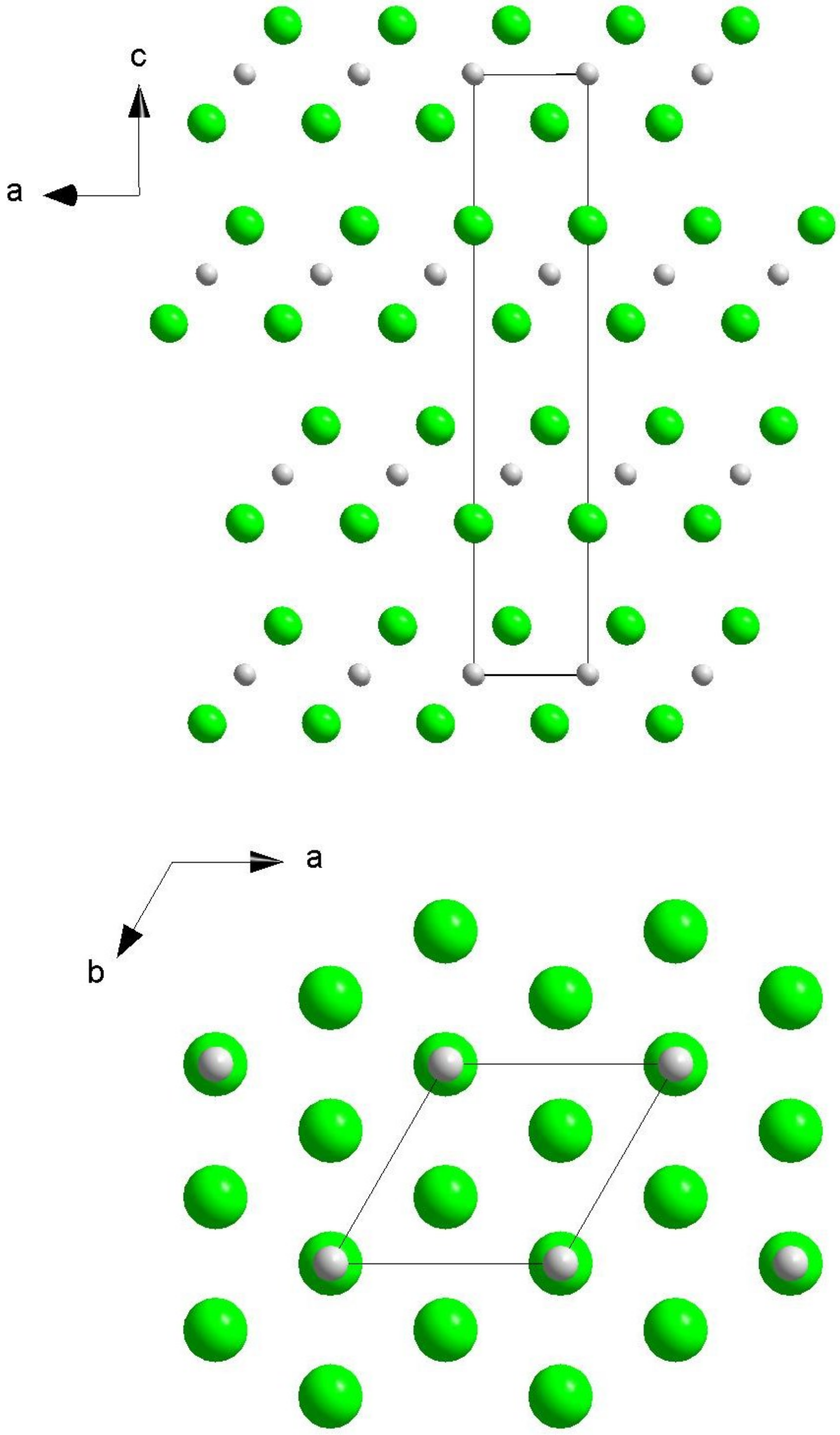

(a)

(b)

Figure S3. Showing the crystallographic packing in $CdCl_2$, the archetypal structure for homoleptic XeF_2 complexes. Cd atoms are shown in grey, Cl are shown in green.

Table S1. CuSb Unit cell dimentions as a function of temperature

**Temperature (K)**	***a* (Å)**	***b* (Å)**	***c* (Å)**	**α (°)**	**β (°)**	**γ (°)**	***V* (Å^3)**
**200**	10.0302(3)	10.0302(3)	22.4539(9)	90	90	120	1956.33(13)
**190**	10.0204(2)	10.0204(2)	22.4318(8)	90	90	120	1950.58(10)
**180**	10.0122(3)	10.0122(3)	22.4147(8)	90	90	120	1945.91(14)
**170**	9.4864(13)	13.7227(17)	19.880(3)	89.503(4)	88.661(4)	87.490(4)	2584.7(6)
**160**	9.4695(6)	13.7090(9)	19.8694(13)	89.558(2)	88.666(2)	87.536(2)	2576.3(3)
**150**	9.4613(7)	13.7006(9)	19.8498(13)	89.545(2)	88.644(2)	87.545(2)	2569.9(3)
**140**	9.4527(7)	13.6910(10)	19.8294(14)	89.534(2)	88.634(2)	87.556(2)	2563.1(3)
**130**	9.4468(7)	13.6851(10)	19.8167(14)	89.512(2)	88.611(2)	87.575(2)	2558.8(3)
**120**	9.4381(6)	13.6755(9)	19.8035(13)	89.4882(19)	88.579(2)	87.596(2)	2552.9(3)
**110**	9.4303(7)	13.6668(9)	19.7902(13)	89.479(2)	88.570(2)	87.608(2)	2547.5(3)
**100**	9.4229(7)	13.6584(9)	19.7796(13)	89.464(2)	88.550(2)	87.633(2)	2542.6(3)
**90**	9.4153(6)	13.6495(9)	19.7700(13)	89.443(2)	88.524(2)	87.651(2)	2537.7(3)
**80**	9.4079(6)	13.6400(8)	19.7587(11)	89.429(2)	88.503(2)	87.680(2)	2532.5(3)
**30**	9.3460(7)	13.5709(10)	19.6883(15)	89.359(2)	88.429(2)	87.765(2)	2494.2(3)

Table S2. ZnSb Unit cell dimentions as a function of temperature

Temperature (K)	*a* (Å)	*b* (Å)	*c* (Å)	α (°)	β (°)	γ (°)	*V* (Å^3)
**200**	10.0769(3)	10.0769(3)	22.4412(12)	90	90	120	1973.47(16)
**190**	10.0717(5)	10.0717(5)	22.4298(15)	90	90	120	1970.4(2)
**180**	10.0651(6)	10.0651(6)	22.4154(18)	90	90	120	1966.6(3)
**170**	10.0568(6)	10.0568(6)	22.3967(17)	90	90	120	1961.7(2)
**160**	9.4815(9)	13.7051(14)	20.0480(19)	89.509(3)	88.721(3)	86.955(3)	2600.7(4)

Table S3. Distortions in the geometry of the Cu and Zn coordination spheres in the phases reported relative to those in **CuSb-I** and **ZnSb-I** at 200 K. For **CuSb-II** at 170 K the three M−F distances refer to those around Cu1, Cu2 and Cu3; the figure in brackets is the standard uncertainty of the individual distance measurements. CSM = continuous shape measure, the three figures reported for CuSb-II referring to Cu1, Cu2 and Cu3 *etc*.

**Phase**	**Average M–F** (Å)	**CSM *vs* MSbI_200K**<br>**M** = Cu or Zn
**CuSb-I** at 200 K	Cu1 1.9877(19)	0 (by definition)
**CuSb-II** at 170 K	Cu1 2.007<br>Cu2 1.993<br>Cu3 1.992 (0.006)	0.175<br>0.135<br>0.211
**ZnSb-I** at 200 K	Zn1 2.001(5)	0 (by definition)
**ZnSb-II** at 160 K	Zn1 2.009<br>Zn2 2.007<br>Zn3 1.997 (0.005)	0.059<br>0.029<br>0.016
**ZnAs-I** at 100 K	Zn1 1.998<br>Zn2 2.007 (0.011)	0.016<br>0.027
**ZnAs-II** at 0.15 GPa	Zn1 1.995<br>Zn2 1.982 (0.009-0.02)	0.058<br>0.022

Table S4. ZnAs-I Unit cell dimesntions as a function of temperature

Temperature (K)	*a* (Å)	*b* (Å)	*c* (Å)	α (°)	β (°)	γ (°)	*V* (Å^3)
**200**	9.198(4)	10.396(4)	13.291(3)	89.72(3)	89.02(3)	86.33(4)	1268.1(8)
**180**	9.183(3)	10.381(3)	13.274(3)	89.69(2)	88.98(3)	86.37(3)	1262.6(7)
**160**	9.170(3)	10.371(3)	13.257(3)	89.65(2)	89.47(3)	86.41(3)	1258.2(7)
**140**	9.152(2)	10.350(2)	13.240(3)	89.63(2)	89.44(2)	86.43(2)	1251.6(8)
**120**	9.138(2)	10.337(3)	13.225(3)	89.58(2)	89.38(2)	86.47(2)	1246.8(7)
**100**	9.1236(7)	10.3242(9)	13.2086(10)	89.555(3)	89.350(3)	86.509(3)	1241.75(17)
