# Supplementary figures and images for: Phase relationships in homoleptic com­plexes of XeF2
Source: IUCrJ. 2026 May 18;13(Pt 4):420–32. doi: 10.1107/S2052252526003751 (PMC13324652; doi:10.1107/S2052252526003751)

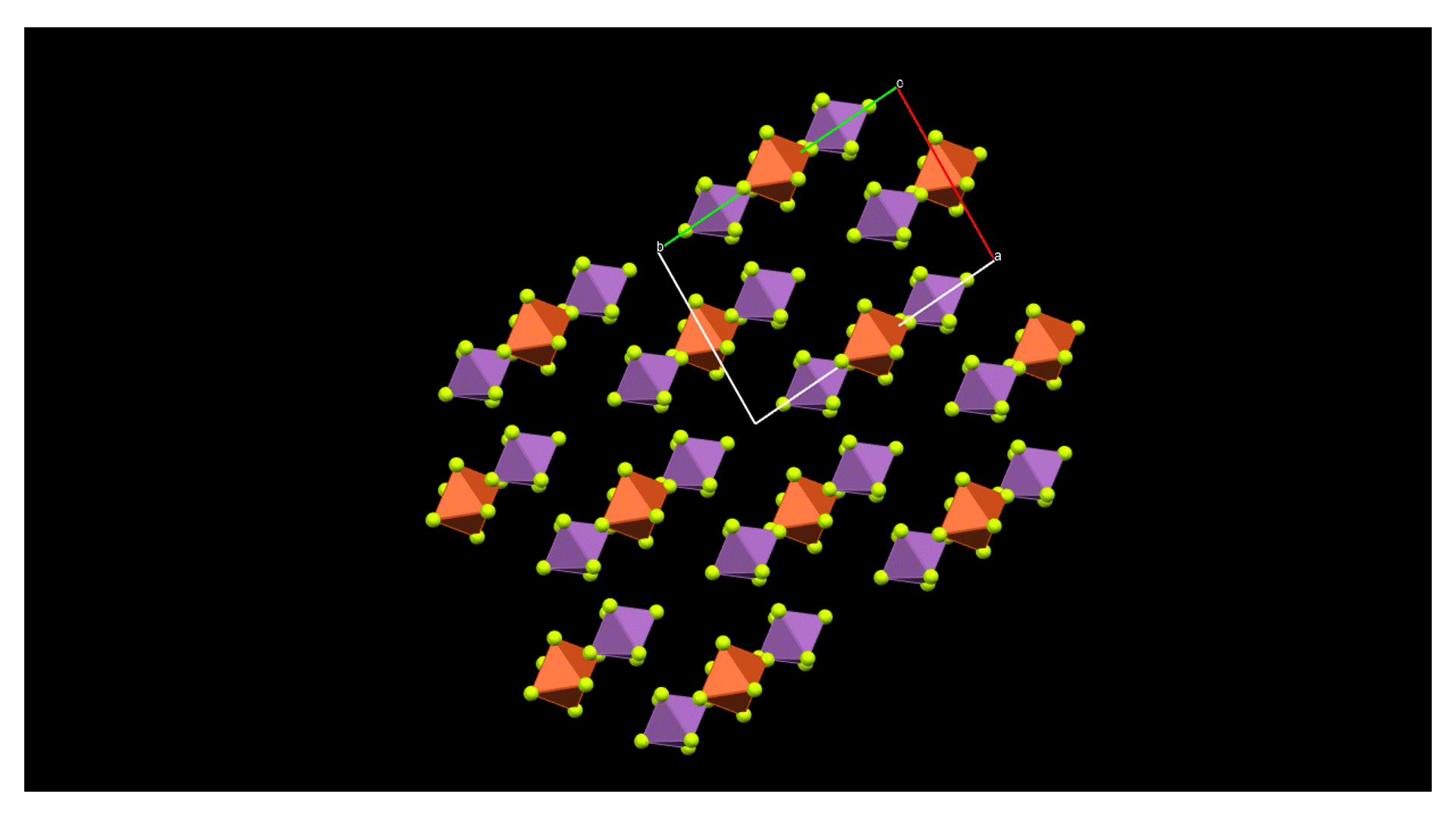

Supplement: Supplementary file 19 [file m-13-00420-sup19.gif]

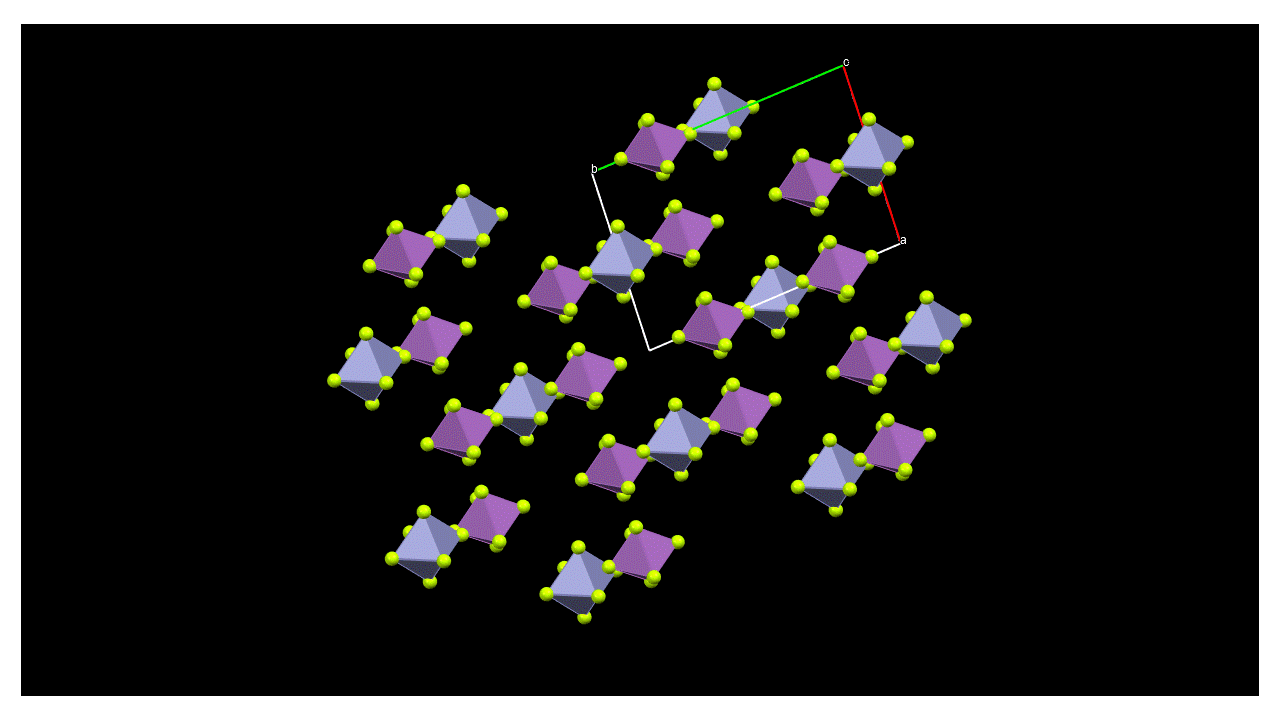

Supplement: Supplementary file 20 [file m-13-00420-sup20.gif]

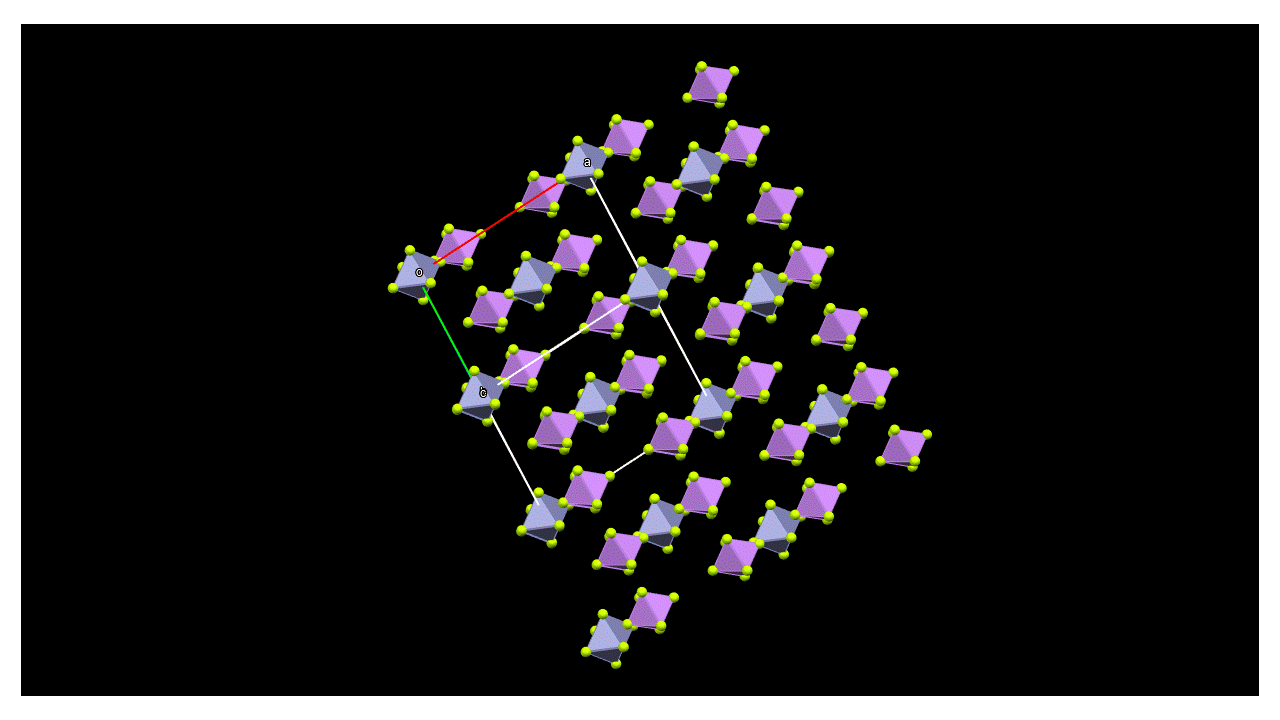

Supplement: Supplementary file 21 [file m-13-00420-sup21.gif]

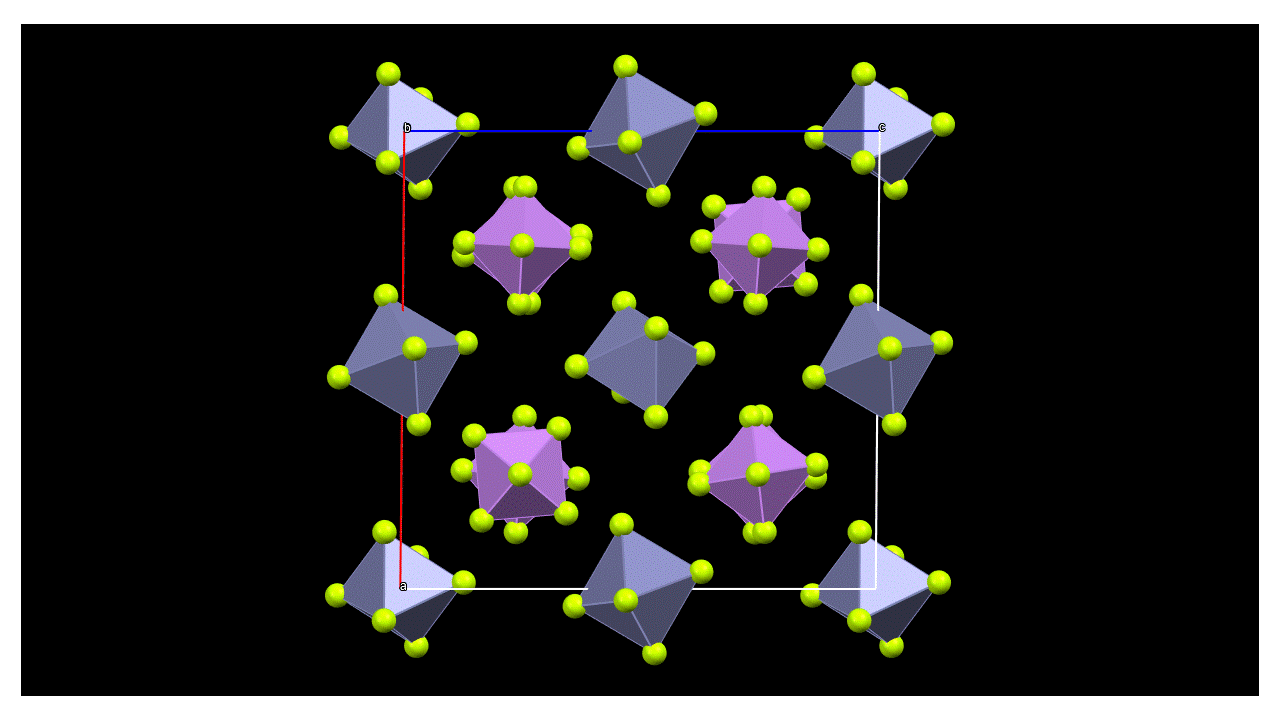

Supplement: Supplementary file 22 [file m-13-00420-sup22.gif]
